# BioDry: An Inexpensive, Low-Power Method to Preserve Aquatic Microbial Biomass at Room Temperature

**DOI:** 10.1371/journal.pone.0144686

**Published:** 2015-12-28

**Authors:** Steven J. Tuorto, Chris M. Brown, Kay D. Bidle, Lora R. McGuinness, Lee J. Kerkhof

**Affiliations:** 1 Department of Marine and Coastal Science, Rutgers University, New Brunswick, New Jersey, United States of America; 2 Environmental Proteomics N.B. Inc, Sackville, New Brunswick, Canada; University of Aveiro, PORTUGAL

## Abstract

This report describes BioDry (patent pending), a method for reliably preserving the biomolecules associated with aquatic microbial biomass samples, without the need of hazardous materials (e.g. liquid nitrogen, preservatives, etc.), freezing, or bulky storage/sampling equipment. Gel electrophoresis analysis of nucleic acid extracts from samples treated in the lab with the BioDry method indicated that molecular integrity was protected in samples stored at room temperature for up to 30 days. Analysis of 16S/18S rRNA genes for presence/absence and relative abundance of microorganisms using both 454-pyrosequencing and TRFLP profiling revealed statistically indistinguishable communities from control samples that were frozen in liquid nitrogen immediately after collection. Seawater and river water biomass samples collected with a portable BioDry “field unit", constructed from off-the-shelf materials and a battery-operated pumping system, also displayed high levels of community *r*RNA preservation, despite a slight decrease in nucleic acid recovery over the course of storage for 30 days. Functional *m*RNA and protein pools from the field samples were also effectively conserved with BioDry, as assessed by respective RT-PCR amplification and western blot of ribulose-1-5-bisphosphate carboxylase/oxygenase. Collectively, these results demonstrate that BioDry can adequately preserve a suite of biomolecules from aquatic biomass at ambient temperatures for up to a month, giving it great potential for high resolution sampling in remote locations or on autonomous platforms where space and power are limited.

## Introduction

Discerning the dynamic processes and interactions of natural microbial communities is key to understanding global biogeochemical cycles. Efforts over the past two decades have revealed that microbial communities are more diverse, complex, and variable than previously appreciated for nearly every environment that has been investigated [1,2]. Furthermore, there is increasing evidence that single-celled populations can exhibit functional variability over small spatio-temporal scales [3–5]. Many studies addressing these issues have relied on the characterization of biomarkers to get a snapshot of the structure and function of microbial communities and the forces controlling biogeochemical activity. In order to determine “who is there, who is active, and when” from environmental samples, researchers often analyze biomolecular components (i.e. DNA, RNA, proteins, etc.) to document the microbes responsible for biogeochemical activity. These studies necessitate that the microbial target biomolecules are adequately preserved for the respective ‘omics’ methodologies (reviewed in [6]). Historically, refrigeration, flash-freezing (liquid nitrogen/dry ice-ethanol), and/or storage at 4 to -196°C has been used to preserve samples until processing in the laboratory. Recently, other methods for preserving and recovering macromolecules such as DNA [7], RNA [8], and proteins [9] have been reported, most of which involve the use of a liquid preservative. The most common storage material, RNALater, has been independently shown to preserve both nucleic acids and proteins [4,7–9]. However, these preservatives often require a clean-up step, and the protection efficacy varies depending on the pre- and post-preservation protocols. The best reported results for certain biomolecules often requires some combination of refrigeration, flash-freezing, and/or storage at -80°C for periods longer than a week (manufacturer’s recommendation), which casts some doubt on the need for a preservative at all if the samples are stored frozen.

In this study, we present an inexpensive, alternative preservation technique, called "BioDry", for reliably protecting aquatic microbial biomass samples from degradation without the need of freezing or bulky storage/sampling equipment. The suitability of the BioDry method to preserve aquatic microbial biomass was determined by comparing the detection of biomolecules from samples that were preserved and stored over a time course with controls that were immediately frozen at liquid nitrogen temperatures and stored at -80°C after collection. The method was first verified in the laboratory using aquarium samples and an apparatus requiring a 120 volt power supply. Later, a portable version of BioDry preservation was field tested employing a commercially available, battery operated air compressor on natural samples. Nucleic acid integrity was analyzed by generating community profiles using both 454 pyrosequencing and TRFLP analyses. Additionally, the efficacy of BioDry preservation for *m*RNA and its associated protein was assessed by ribulose-1,5-bisphosphate carboxylase/oxygenase (RuBisCo) RT-PCR amplification of transcripts and western blot analysis respectively. Our results demonstrate that "BioDried" samples stored at room temperature for up to 30 d (lab tests) or 10 d (field tests) were virtually indistinguishable from the samples immediately placed at liquid nitrogen temperatures after collection. Our data suggest that BioDry can provide a low-cost, portable, robust approach for aquatic sampling in remote environments lacking significant infrastructure. Furthermore, it is ideal for incorporation into autonomous platforms where power and weight requirements significantly limit the types of sample collection and the methods of preservation.

## Materials and Methods

Aquatic biomass samples were obtained under a scientific collecting permit (MFA-SCP No: 1409) from the New Jersey Department of Environmental Protection.

### In-Lab BioDry Apparatus and Sampling Procedure

BioDrying was performed using a modified 50 ml centrifuge tube containing Drierite, an in-line air flow meter, and an AC powered vacuum pump, all of which were adapted to recirculate desiccated air over sample filters (S1 Fig). To obtain microbial biomass, each aquatic sample was collected onto a 0.2 μm Supor filter using a Swinnex filter holder (S2A Fig). After the sample filtration was complete, any remaining water was manually forced through the filter using a separate syringe to facilitate BioDrying. Each filter was then transferred to its own pre-prepared filter holder constructed from a clipped, sterile, microcentrifuge tube and a small piece of tubing (S3A–S3B Fig) that was pre-washed in detergent/bleach and successively rinsed with DI and sterile water. The filter holder was connected to the pump (S1 Fig), and desiccated air was allowed to flow over the filter for 10–20 min (see below). The filter holder was then closed at both ends (S3C Fig), and stored in a desiccant chamber until processing.

On several different occasions, preliminary quality control tests were performed to assess the effects of the “in-lab" BioDry procedure on nucleic acid recovery, down-stream molecular protocols, and for the possibility of contamination from the desiccating airstream. For nucleic acid recovery, un-incubated seawater samples (~150 ml) from coral reef aquaria were collected onto filters and either immediately frozen at liquid N_2_ temperatures, or preserved as described above, and then immediately frozen. For assessing BioDry sample’s integrity for molecular protocols and susceptibility to contamination, replicate seawater filters were prepared and preserved as described above, while a second set of “air-blank” filters were subjected to the desiccation process only, then flash frozen. Both treatments were stored at -80°C until they could be extracted for total nucleic acids. Nucleic acid yield was determined by agarose gel and the integrity of nucleic acids and presence of contamination from the air stream was verified via PCR amplification (see nucleic acid analysis below).

### Portable BioDry Apparatus and Sampling Procedure

The portable BioDry apparatus consisted of a Ryobi 18-volt One Plus cordless air compressor with a One Plus 18V lithium ion battery, Tygon and vacuum tubing, three valves (for air diversion & pressure relief), two 60 cc syringes, 0.2 μm nylon syringe filters (Millipore), and various fittings to ensure proper seals in the plumbing (S4 Fig). The high-pressure outlet of the compressor was connected so the airstream could be diverted directly into either the back of a collection syringe containing a water sample for filtration, or a preservation syringe which contained Drierite for sample desiccation. The air flow rate exiting the desiccation syringe was determined to be ~50 square cubic feet h^-1^ (SCFH) using an air-flow meter.

An aquatic sample was concentrated onto the 0.2 μm syringe filter via the sample collection syringe (S2B and S4 Figs). The syringe filter was then transferred to an empty syringe and any remaining water was manually forced through the filter as above. The syringe filter housing was then partially cleaved (≈ 1/2 way through) just above the plane of the filter, using a ratcheting PVC cutter (S5A Fig). This allowed free airflow over the filter and biomass. The syringe filter was then moved to the desiccant syringe (S5B Fig) and the desiccated air was allowed to flow through the filter housing for 8 min.

### BioDry Method Verification

Seawater from an ocean-reef aquarium was collected and incubated with 0.01% Zobell’s medium overnight to generate microbial samples for preservation. In this trial, 30 ml of incubated seawater was collected by 50 ml syringe and Swinnex (described above) for each treatment. The samples were repetitively sampled from the incubated seawater water which was kept homogenous using a stir-bar, and the syringes were rinsed 3x with sterile water in between samples. First, duplicate samples were collected on filters, snap-frozen (liquid N_2_), and then stored at -80°C to serve as T_0_ controls. For BioDry assessment, replicate filters (n = 8) were used to collect biomass and BioDried using the in-lab procedure (see above) at the full air-flow capability of the pumping apparatus (≈ 90 SCFH) for either 10 (n = 4) or 20 (n = 4) min. The procedure was then repeated (e.g. 10 (n = 4) or 20 (n = 4) min) with a pinch valve on the tubing to restrict the air-flow to ≈ 50 SCFH (chosen as the equivalent to the portable apparatus air flow). These served to assess the procedure for both high and low air-flow rates, as well as for shorter and longer BioDrying durations. Two of each of these treatments (2 x high/low air-flow x long/short BioDrying time; n = 8) were stored in individual desiccant chambers (to be processed at 15 and 30 d). Two additional filters were made and BioDried at high-flow for 10 min, and stored in a separate desiccant chamber. After 15 d (T_15_), the chamber storing the two extra filters was opened, and the filter holders were exposed to ambient air for 2 consecutive days before replacing them in their desiccant chamber. This was done to determine if exposure to ambient humidity triggered degradation of biomolecules, and whether or not constant storage in a desiccation chamber was necessary. Also at this time, all T_15_ filters were transferred to individual 2.0 ml centrifuge tubes, snap-frozen, and placed at -80°C to halt any further sample degradation. After 30 d (T_30_) the sampling process was repeated with the second batch of filters and for the air-exposed filters.

### Assessment of Temperature and Humidity on Preserved Samples

In a separate trial, incubations similar to those described above were repeated to determine if preserved samples were susceptible to temperature and/or humidity effects. Here, 30 ml was collected onto syringe filters and the preservation process was done using the in-lab BioDry apparatus. A set of duplicate control filters and 12 BioDry treatment filters were collected using a drying time of 8 min. Two of the BioDry filters were each placed in one of 6 Qorpak jars. Three of the jars contained DrieRite to maintain dry storage conditions (n = 6). Three jars contained a sponge saturated with distilled water to simulate humid storage conditions (n = 6). A set of both dry-storage and humid-storage jars were placed at 18, 27, and 37°C to determine temperature effects on preservation. After 20 days the syringe filter housings were completely cleaved open, the filters excised with a scalpel, and transferred to 2.0 ml centrifuge tubes and snap-frozen. As degradation is usually observed very quickly via our analyses, it was not deemed necessary to wait 30 days to assess the effects of temperature and humidity. Once transferred and frozen, all filters were stored at -80°C until they could be processed.

### BioDry Field Verification

The BioDry method was tested in the field using the portable apparatus (S4 Fig) on aquatic samples from both a saline and freshwater environment. Samples were individually collected by hand (without homogenization) and preserved on-site both off the coast of Belmar, NJ and from the Delaware River in Hopewell, NJ. For each site triplicate control (i.e. T_0_) samples were collected onto the syringe filters, snap-frozen and stored at -80°C. Replicate BioDry treatment filters (n = 6) were collected, preserved using the portable BioDry apparatus for 8 min (see above), and stored in plastic jars containing DrieRite. A storage time of 10 days was selected since autonomous platforms (e.g. Slocum gliders) typically deploy for comparable time periods. The other time point (30 days) was chosen to directly compare field BioDry results to the laboratory BioDry study. One set of triplicate BioDried filters was excised and snap-frozen after each of the storage time points (T_10_ and T_30_ days). Finally, an additional set of control and BioDried filters (n = 3) was collected and processed at T_10_ days for assessment of the BioDry method to protect proteins from aquatic microbial biomass.

### Nucleic Acid Analysis

Total nucleic acids were purified from samples using a modified phenol/chloroform/isoamyl alcohol protocol [10,11]. Briefly, filters underwent 5 freeze-thaws followed by a 10 min lysozyme digestion. Samples were then subjected to phenol/chloroform/isoamyl alcohol extraction, ethanol precipitation, and re-suspension in DEPC-treated water. The extracts were run on 1% agarose gels for quality control and quantitation by ethidium bromide fluorescent intensity and image analysis [12]. Genomic DNA quality was also assessed using two analytical methods for comparing microbial community composition. First, genomic DNA from pooled sample extracts (n = 3 each of controls, T_15_, and T_30_ treatments) was sent for 454-pyrosequencing of the bacterial community by Mr. DNA (16S rRNA gene sequencing; approx. 30,000 reads/sample; www.mrdnalab.com, MR DNA, Shallowater, TX). Small subunit ribosomal *r*RNA sequences were generated using a Roche 454 FLX titanium instrument following the manufacturer’s guidelines. Barcodes, primers, short sequences < 200bp, sequences with ambiguous base calls, and sequences with homopolymer runs exceeding 6 bp were removed. The data was also denoised, chimeras were removed, and operational taxonomic units were defined after removal of singleton sequences by clustering at 3% divergence (97% similarity) as previously described [13]. These OTUs were then classified against a curated GreenGenes database by BLASTn [14]. All sequences were submitted as uncultured bacteria to GenBank’s BioSample database (Bioproject PRJNA278276). Additionally, 16S rRNA gene community TRFLP fingerprints were generated for the bacterial community from both pooled DNA (laboratory tests), as well as from the ribosomes of each individual replicate (laboratory and field tests) from each treatment as previously described [5,10,11].

For the seawater and riverine field samples, RNA bands for each extract were excised from an agarose gel and purified with an RNaid kit (MP Biomedicals, Solon, OH, USA). The SSU ribosomes were amplified with the Titan One Tube RT-PCR kit (Roche Diagnostics, Indianapolis, IN, USA) using either bacterial, eukaryotic, or archaeal primers. Equal volumes of either DNA or dilute RNA (10^−3^ for bacterial and eukaryotic reactions; 10^−2^ for archaeal reactions) were amplified for comparison of preservation treatments and controls. For RT-PCR reactions, bacterial reaction mixtures contained 20 pmol of the universal primers 27 Forward (5’-AGA GTT TGA TCC TGG CTC AG-3’; labeled with the fluorochrome 6’-FAM) and 1100 Reverse (5’-AGG GTT GCG CTC GTT G-3’) per reaction. The PCR amplification parameters were 50°C for a 30 min reverse transcription step and then 27 cycles of 94°C for 10 s, 57°C for 30 s, and 68°C for 2 min with a final extension at 68°C for 7 min. Eukaryotic reaction mixtures contained 20 pmol of 18 Forward (5’-ACC TGG TTG ATC CTG CCA G-3’; also labeled with 6’-FAM) and 516 Reverse (5’-ACC AGA CTT GCC CTC C-3’) per reaction and ran for 27 cycle at 94°C for 30 s, 56°C for 30 s, and 68°C for 1 min. Archaeal reaction mixtures contained 20 pmol of 21 Forward (5’-TTC CGG TTG ATC CYG CCG GA-3’; also labeled with 6’-FAM) and 958 Reverse (5’-YCC GGC GTT GAM TCC AAT T-3’) per reaction with 35 cycles at 94°C for 30 s, 55°C for 30 s, and 72°C for 1 min 20 s. Control reactions (DNA/contamination) were run without a reverse transcription step and with an extension temperature of 72°C.

To generate TRFLP fingerprints, equal masses of fluorescent PCR product (20 ng) were digested with *Mnl* I (bacterial and archaeal) or *Hae* III (eukaryotic) in 20 μl reactions for 6 h at 37°C. The digests were precipitated and re-suspended in 19.7 μl of deionized formamide with 0.3 μl of ROX 500 standard and analyzed on an ABI 310 Genetic analyzer with peak detection/quantification employing Genescan software (Applied Biosystems, Foster City, CA). All TRFLP profiles generated can be viewed in supplemental materials (S8–S14 Figs)

Both the 454-pyrosequence data (pooled bacterial DNA only) and TRFLP profiles (pooled bacterial DNA & replicate bacterial/eukaryotic/archael RNA) of preserved samples were compared to T_0_ frozen controls for presence/absence and relative proportion of operational taxonomic units (OTUs). A pairwise, average Sorensen’s index measure (*C*_*S*_) was used to assess sample reproducibility (i.e. similarity between replicates) and preservation between treatments (i.e. similarity between T_0_ and either T_10_, T_15_, or T_30_), and the percent contribution of OTUs in controls were plotted against those in preserved samples to determine if the community’s relative proportion was conserved. Additionally, a Bray-Curtis similarity index was generated for all 454 and TRFLP analyses to assess sample reproducibility and conservation when employing the BioDry method (S3–S10 Tables).

### mRNA and Protein Analysis

The preservation of clade specific *m*RNA transcripts responsible for the production of RuBisCo in BioDried samples was also determined. First, DNA was removed from aliquots of the T_0_, T_10_, and T_30_ seawater extracts using an Ambion TURBO DNA-*free* ^™^ kit (Life Technologies, Carlsbad, CA, USA). Diatom- and haptophyte-specific RbcL transcripts were amplified from the RNA sample with the Titan One Tube RT-PCR kit (Roche Diagnostics, Indianapolis, IN, USA) as in John et al., 2007 [15]. Briefly, 2 μl of template was used to amplify diatom transcripts using the forward primer (5’-GAT GAT GAR AAY ATT AAC TCW), and the reverse primer (5’-TAW GAA CCT TTW ACT TCW CC). The thermocycler parameters were: 45°C hold for 30 m, 95°C hold for 10 m, then 40 cycles of 95°C for 20 s, 52°C for 60 s, 72°C for 60 s, followed by a final extension step for 7 m at 72°C. Haptophyte transcripts were amplified using 2 μl of template and the forward primer (5’-GWG AGC GTT TCC TTT ACT C), and reverse primer (5’-GCA CGY TCR TAC ATR TCT TC) with the following thermocycler parameters: 45°C hold for 30 m, 95°C hold for 10 m, then 40 cycles of 95°C for 20 s, 54°C for 60 s, 72°C for 60 s, followed by a final extension step for 7 m at 60°C. A no-RT (DNA/contamination) control was performed in a separate set of PCR reactions with 2 μl aliquots of the RNA extract using the same parameters as above, but without the reverse transcription step. Presence of *m*RNA transcripts was verified on 1% agarose gels by ethidium bromide fluorescent intensity and image analysis.

Lastly, samples taken for protein analysis were extracted in an LDS extraction buffer via sonication as in Brown et al., 2008 [16]. Briefly, 200 μl of extraction buffer (140mM Tris base, 105mM Tris-HCl, 0.5mM EDTA, 2% LDS, 10% glycerol, with 0.1mg/ml protease inhibitor) was added to the filters and immediately flash-frozen in liquid N_2_. The filters were then sonicated with a microtip (Misonix, Farmingdale, NY; power setting of 2–3) for approximately 30s or until samples were beginning to thaw. The sample was then immediately refrozen. Two to three more rounds of sonication/freezing followed, being careful not to let heat accumulate in the sample. Following disruption, the samples were centrifuged to pellet insoluble material, and the supernatant was transferred to a new tube and stored at -20°C.

Total protein content was determined for all samples using a Bio-Rad DC-assay (Bio-Rad Labs, Hercules, CA, USA) with bovine gamma globulin as a standard. Specific protein integrity was assessed by western blot using a ribulose-1,5-bisphosphate carboxylase/oxygenase (RuBisCo) large subunit (RbcL) antibody. Briefly, after separation of the proteins by polyacrylamide electrophoresis, (200 V for 40 m) the gel was blocked for 1 h in 2% ECL blocking reagent (GE Amersham) in TBS-T (0.1% Tween-20). Proteins were transferred to PVDF (100V for 30 m) then incubated with the RbcL antibody (Agrisera) at a dilution of 1:40000 for 1 h at room temperature with agitation. After washing 1 x 15 m, then 3 x 5 m in TBS-T, the membrane was incubated with goat anti-rabbit horse radish peroxidase secondary antibody at a dilution of 1:20000 for 1 h at room temperature. The blot was then washed as above, developed in ECL Select (GE Amersham) according to the manufacturer’s instructions, imaged with a BioRad Chemidoc MP (Bio-Rad, Hercules, CA) and RbcL bands quantified relative to quantitative calibration standards (Environmental Proteomics) using Image Lab software to determine band volumes.

### Statistical Analysis

A Two-Sample t-Test was used for any comparison of means at α = 0.05. All data and statistical analysis was done using either XLStat (Addinsoft SARL) in Excel 2010 (Microsoft Corporation) or SPSS Statistics v21 (IBM Corporation).

## Results

### BioDry Method Verification

In several sets of distinct preliminary tests, both the efficacy of the BioDry procedure on yield and integrity of biomass samples, as well as susceptibility of samples to contamination from the desiccation process were assessed via agarose gel and PCR amplification. There was a slight, but repeatable decrease in DNA yield after samples were treated with BioDry, but no sign of degradation or shearing of the nucleic acids was apparent (representative results in S6A Fig). In all molecular integrity/contamination tests, PCR amplicon was detected for the seawater biomass samples, and no PCR amplicon was detected for the “air-blank” filters (representative results in S6B Fig). From this it was concluded that sterile-filtration of the desiccating air stream was not necessary, and would only result in an adverse reduction in air flow.

**Fig 1:**
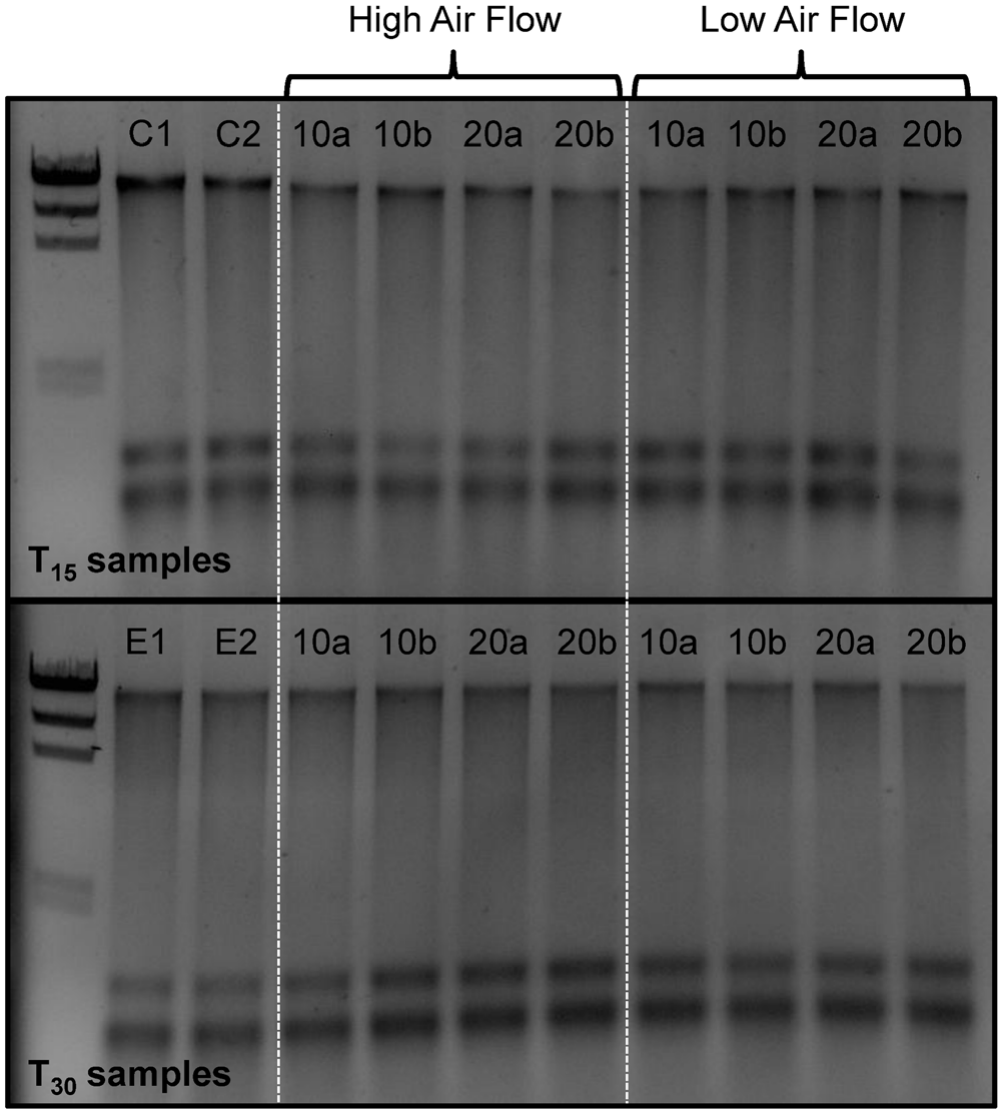
Agarose gel of DNA/RNA extracts from the laboratory samples preserved with BioDry. The frozen controls (C1 and 2) are followed by samples that were preserved at either high or low air-flow rates, for 10 (lanes 10a and b) or 20 (lanes 20a and b) min, and then stored at room temperature in a desiccant chamber for T_15_ or T_30_ days (top & bottom images respectively). Lanes E1 and 2 in the bottom image represent the filters whose holders were exposed to ambient air for 2 days.

The initial BioDry experiment was designed to assess nucleic acid integrity as a function of desiccated air flow-rate and exposure time by visualization of nucleic acids on agarose gels (Fig 1). Quantification of DNA in preserved compared to frozen controls demonstrated a slight decrease in yield, while RNA mass remained largely the same between preserved and control samples (Table 1). This is consistent with all preliminary QC tests (see above; S6 Fig). When comparing the effect of the treatments on samples post-preservation, there was no observable difference between preserved replicates with respect to differential air-flows (DNA: *t*(14) = 1.18, *p* = 0.25; RNA: *t*(14) = 1.05, *p* = 0.31), drying times (DNA: *t*(14) = 0.11, *p* = 0.91; RNA: *t*(14) = 0.67, *p* = 0.51), or storage time (DNA only: *t*(14) = 1.63, *p* = 0.12). For this reason the values for each time point given in Table 1 are averages of all treatments. A second experiment was designed to assess storage temperature and humidity on BioDry preservation (S7 Fig). With the exception of the loss of RNA yield in one of the 37° replicates, the samples which were stored dry contained intact nucleic acids with mean yield DNA (9 ± 3 ng μl^-1^) and RNA (27 ± 6 ng μl^-1^) values that were not statistically different from the control’s DNA or RNA (7 ± 2 & 33 ± 3 respectively; *t*(4) = 1.18, *p* = 0.30 & *t*(3) = 1.82, *p* = 0.17 respectively). Conversely, both the DNA and RNA in the BioDried samples that were stored under humid conditions were visibly degraded (S7 Fig).

**Table 1:** Biomolecule Quantification (±SD)–Nucleic acid quantity (ng/μl) from laboratory tests in controls vs. BioDried samples (15d or 30d); Field tests of nucleic acids (ng/μl), total protein content (ng/μl), and RuBisCo large subunit (fmol/μg of protein by Western Analysis) from BioDried samples (10d or 30d; na = not available).

Test	Biomolecule	Control	15 d	30 d
Lab Tests	DNA	6 +/- 1	3 +/- 1	3 +/- 1
	RNA	5 +/- 1	5 +/- 1	7 +/- 1
		**Control**	**10 d**	**30 d**
Field Tests	DNA (sw)	8 +/- 2	4 +/- 1	4 +/- 1
	RNA (sw)	6 +/- 1	6 +/- 1	4 +/- 1
	DNA (riv)	6 +/- 1	1 +/- 1	0 +/- 0
	RNA (riv)	3 +/- 0	5 +/- 1	0 +/- 0
	Total Protein (sw)	114 +/- 5	172 +/- 5	na
	RbcL (sw)	84 +/- 6	70 +/- 12	na

Community analysis by both 454-pyrosequencing and TRFLP was used to assess whether there was preferential degradation of DNA and/or RNA during BioDry preservation. The number of bacterial operational taxonomic units (OTUs) detected in the 454-pyrosequencing of the bacterial 16S *r*RNA genes in DNA indicated nearly identical numbers of OTUs and a high level of similarity in OTU identity between preserved and control samples, as measured by the Sorenson’s similarity index (*C*_*s*_) (Table 2). This was based on approximately 30, 35, and 21 x 10^3^ sequence reads that passed the quality filters for the T_0_, T_15_, and T_30_ samples respectively. Closest match classifications revealed that nearly all dominant organisms were preserved through 30 days, and that they were represented by a broad range of species across the Proteobacteria, Actinobacteria, Bacteroidetes/Flavobacteria, and the GN02 candidate-division Phyla (S2 Table). The number of OTUs measured by DNA-TRFLP profiling (S8 Fig) decreased slightly from T_0_ and T_15_ to T_30_, but there was a high level of similarity in the community OTU identity (see *C*_*s*_ values; Table 2). Likewise, the RNA-TRFLP replicates were highly repeatable (S9 Fig). Total number of OTUs detected in the T_15_ or T_30_ samples were not significantly different (*t*(8) = 0.36, *p* = 0.72 & *t*(8) = 0.50, *p* = 0.63 respectively), and OTUs similarities remained high compared to the T_0_ samples (Table 2).

**Table 2:** OTU and Sorensen’s similarity index measure (*C*_*S*_) for the laboratory verification tests. The average number of bacterial OTUs (± SD) are indicated. (There is no SD for the pooled DNA-TRFLP or 454 samples; for RNA-TRFLP the n = 2 for controls and n = 8 for preserved; na = not available).

	Bacterial DNA-TRFLP		Bacterial RNA-TRFLP		Bacterial DNA-454	
	OTUs	*C*_*s*_	OTUs	*C*_*s*_	OTUs	*C*_*s*_
Control	43	na	25 +/- 0	na	131	na
15 d	42	0.89	26 +/- 2	0.92	132	0.91
30 d	34	0.83	26 +/- 2	0.82	132	0.86

**Fig 2:**
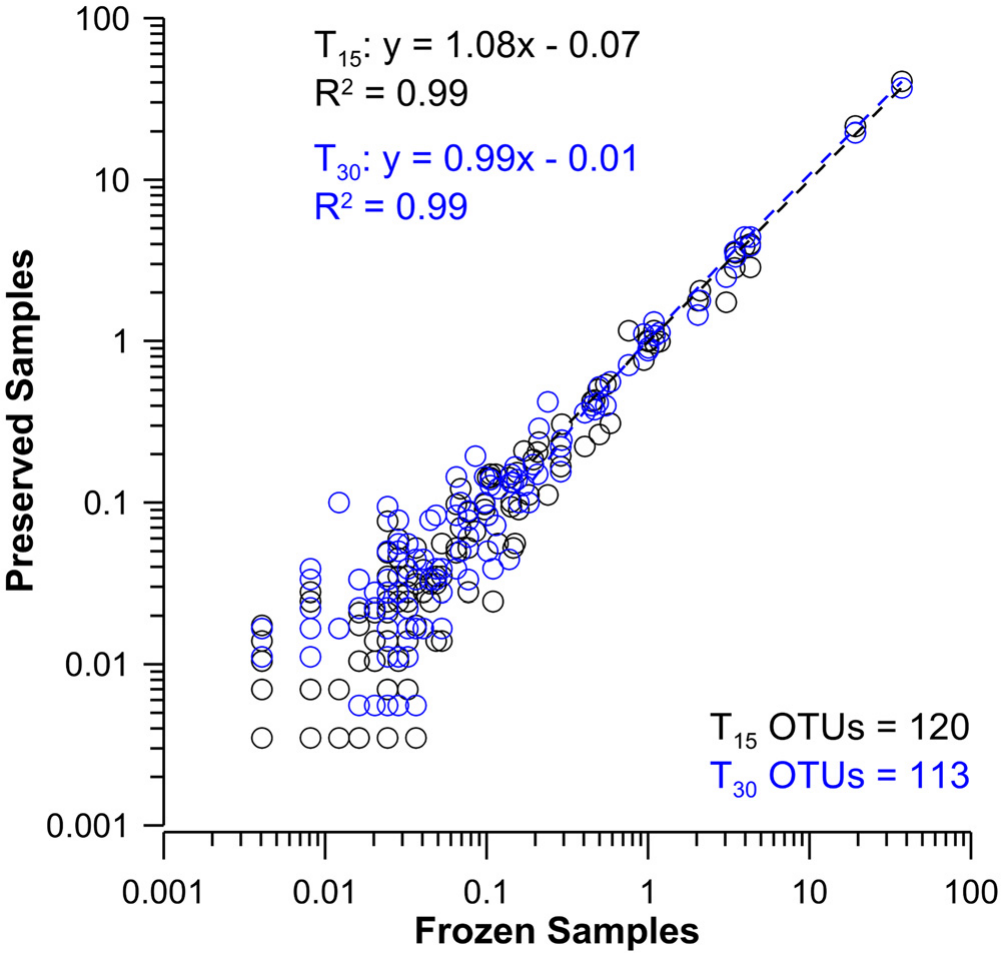
Comparison of species relative percent contributions in frozen controls with samples preserved using BioDry, as determined by 454-pyrosequencing. Results shown for the comparison of DNA samples (n = 3 per treatment) from BioDried samples that were stored for T_15_ and T_30_ d (black and blue profiles respectively) with frozen/control samples. Values represent only those peaks present in more than one replicate for the treatments being compared; the number of OTUs used for each analysis is indicated at the bottom-right of each graph; see S2 Table for taxonomy and relative contributions.

**Fig 3:**
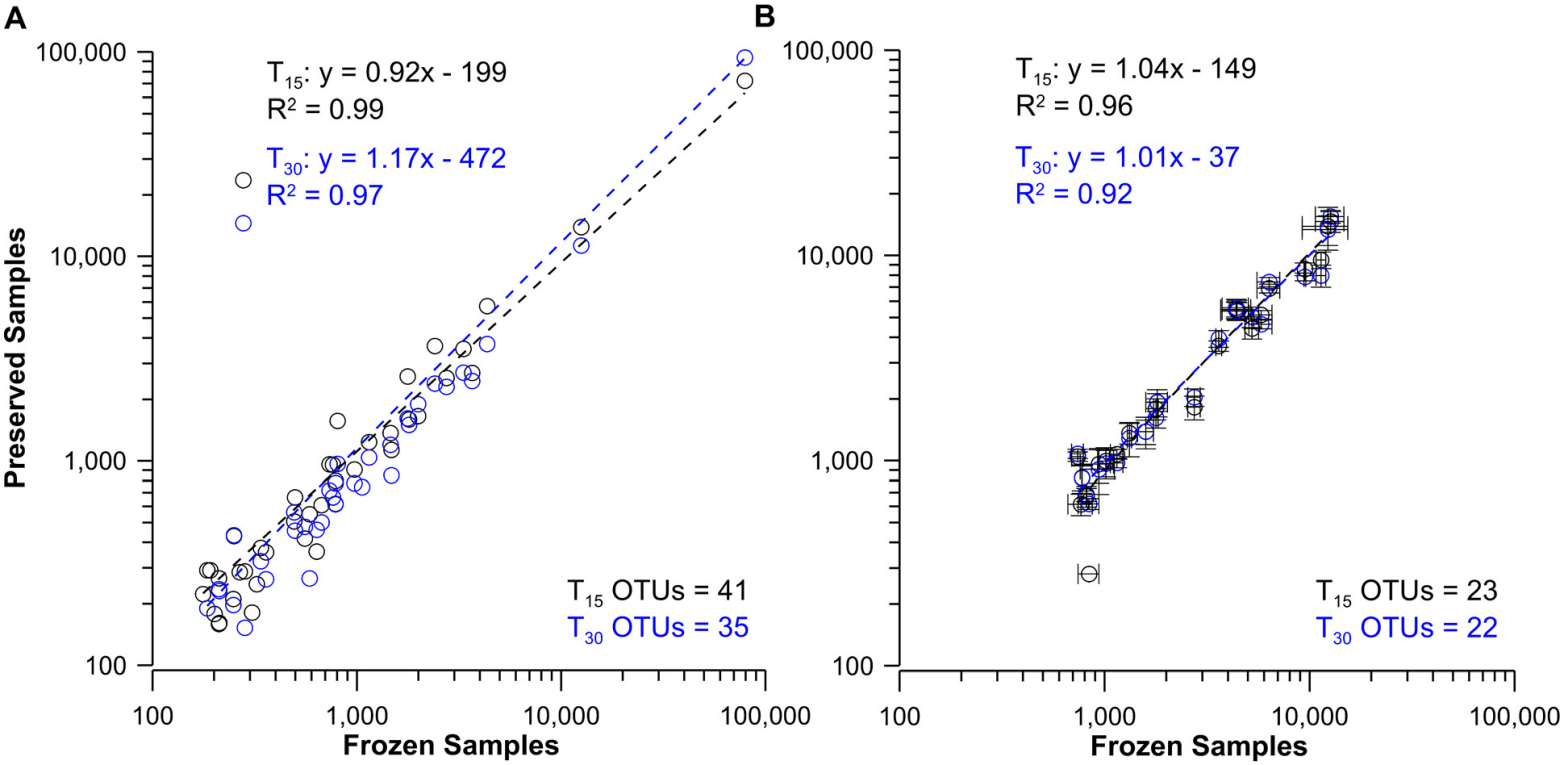
Comparison of frozen control and preserved bacterial DNA and RNA-TRFLP peak areas from laboratory samples preserved with BioDry. Results shown for the comparison of pooled DNA samples (A, n = 3 per treatment) and average ribosomes of all replicates (B, n = 8 ± SD) from BioDried samples that were stored for T_15_ and T_30_ d (black and blue profiles respectively) with frozen samples (controls; n = 2). Values represent only those peaks present in more than one replicate for the treatments being compared; the number of OTUs used for each analysis is indicated at the bottom-right of each graph.

A high level of preservation for both DNA and RNA was observed when the relative proportion of the OTUs present in the T_15_ and T_30_ samples were plotted against the T_0_ samples. The results from both the 454-pyrosequencing (Fig 2) and TRFLP methods (Fig 3) indicate a nearly 1:1 relationships for all comparisons (with all r^2^ ≥ 0.92). Conservation of the community structure between T_0_ and all preservation time points was also apparent in Bray-Curtis similarity indices (S2 and S3 Tables). These showed collective similarities of 0.947 (±0.016), 0.882 (±0.024), 0.858 (±0.06) for the 454 sequence, as well as the DNA and RNA TRFLP analyses respectively (S3–S5 Tables). The pyrosequencing data from frozen and BioDry samples (S2 Table) indicated coefficients of variation for the marine genera representing 50–0.5% of the community differed by 11±7%, the 0.5–0.05% proportion differed by 28±16%, and the 0.05–0.005% proportion differed by 66±34%. Collectively, these data demonstrate the BioDry method is capable of preserving both the diversity and the relative abundance of the nucleic acids of the aquatic microbial community for extended periods of time.

### BioDry Field Verification

**Fig 4:**
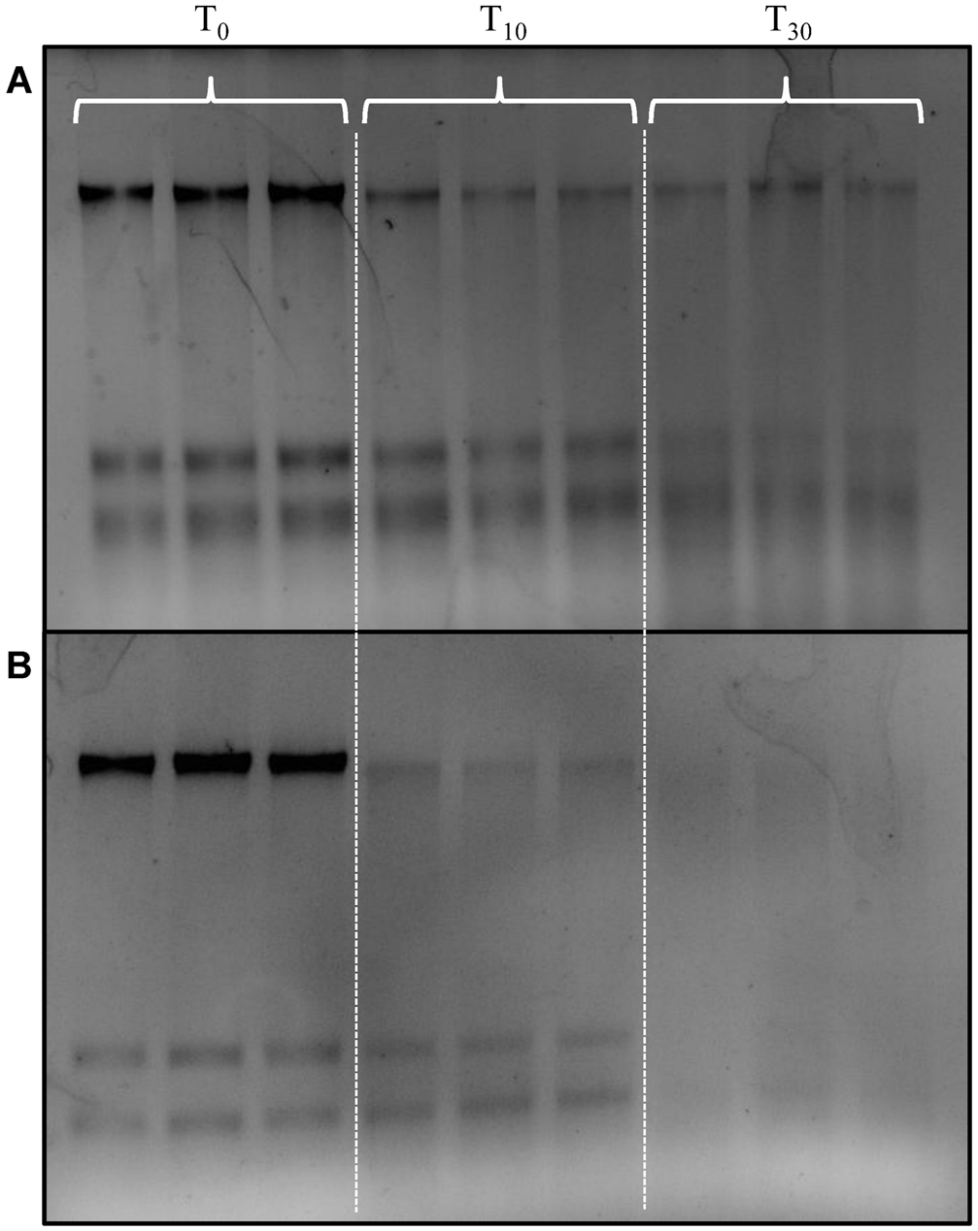
Agarose gel of total nucleic acid extracts from seawater (A) and river water (B) field samples. Samples were either flash frozen in liquid nitrogen and stored frozen (T_0_ Control) or preserved using the portable BioDry apparatus and stored for either T_15_ or T_30_ days at room temperature.

In order to determine if the preservation method was effective on field samples, the aquatic microbial communities from seawater and riverine waters were collected and preserved using the portable BioDry field apparatus (see Materials and Methods; S4 Fig). Agarose gel analysis of T_10_ and T_30_ samples demonstrated a gradual loss in nucleic acid yield with time compared to controls for both types of natural samples (Fig 4). While quantification showed DNA yield decreased from T_0_- T_30_ in both types of aquatic samples, the RNA yields did not indicate loss until T_30_ in the seawater samples (Table 1). Unfortunately, both DNA and RNA yields dropped to below detection by T_30_ in the river water samples (Fig 4; Table 1).

Bacterial, eukaryotic, and archaeal profiles generated by RT-TRFLP (S10–S14 Figs) from RNA in preserved field samples indicated all domains of life could be assayed and showed resulting patterns similar to those of the lab samples. For example, for seawater the average number of bacterial and eukaryotic OTUs detected (Table 3) was not significantly different between T_0_ controls and the BioDried T_10_ (bacterial: *t*(2) = 0.39, *p* = 0.72; eukaryotic: *t*(2) = 2, *p* = 0.18) or T_30_ samples (bacterial: *t*(2) = 1.94; *p* = 0.19; eukaryotic: *t*(2) = 2.33, *p* = 0.14). A similar pattern was observed in the riverine samples, with the average number of bacterial and eukaryotic OTUs detected (Table 3) in frozen T_0_ samples not being significantly different from either the T_10_ (bacterial: *t*(4) = 1.06, *p* = 0.35; eukaryotic: *t*(4) = 1.49, *p* = 0.21) or T_30_ samples (bacterial: *t*(3) = 0.87; *p* = 0.45; eukaryotic: *t*(3) = 1.19, *p* = 0.32). With regards to similarity, however, there was a marked decrease in *C*_*s*_ values for most T_30_ OTU identities (Table 3) which was attributed to the decline in nucleic acid yields at that time point (Fig 4; Table 1). This was also observed in the Bray-Curtis Similarity indices generated for the the bacterial and eukaryotic communities from both sample types. The seawater bacterial and eukaryotic communities showed a decrease in similarity only after 30 days (S6 and S7 Tables), while the river communities showed a general decrease in similarity with time (S8 and S9 Tables).

**Table 3:** OTUs and Sorensen’s similarity index measure (*C*_*S*_) for the field verification tests. The average number of bacterial, eukaryotic, and archaea OTUs (± SD) from the RNA-TRFLP profiles are indicated; (n = 3 for each treatment; na = not available).

		Bacteria		Eukaryotes		Archaea	
	Treatment	OTUs	*C*_*s*_	OTUs	*C*_*s*_	OTUs	*C*_*s*_
Seawater	Control	34 +/- 4	na	20 +/- 1	na	34 +/- 1	na
	10 d	33 +/- 0	0.84	20 +/- 1	0.84	26 +/- 7	0.54
	30 d	27 +/- 2	0.55	25 +/- 2	0.69	43 +/- 6	0.43
Riverine	Control	23 +/- 1	na	28 +/- 2	na	na	na
	10 d	22 +/- 0	0.78	26 +/- 1	0.75	na	na
	30 d	19 +/- 8	0.44	25 +/- 4	0.72	na	na

The number of archaeal ribosomal OTUs in seawater samples were found to be variable between the T_0_, T_10_, and T_30_ treatments, with the highest number of detected OTUs at T_30_. Additionally, archaea were highly heterogeneous between replicates and time points, as attested by both low *Cs* values (Table 3) and Bray-Curtis similarity measures (S10 Table). Approximately 30% of OTUs detected within any given sample was represented by unique peaks, i.e. not found in any other sample at any time point. Archaea were below PCR detection limits altogether in the riverine samples.

**Fig 5:**
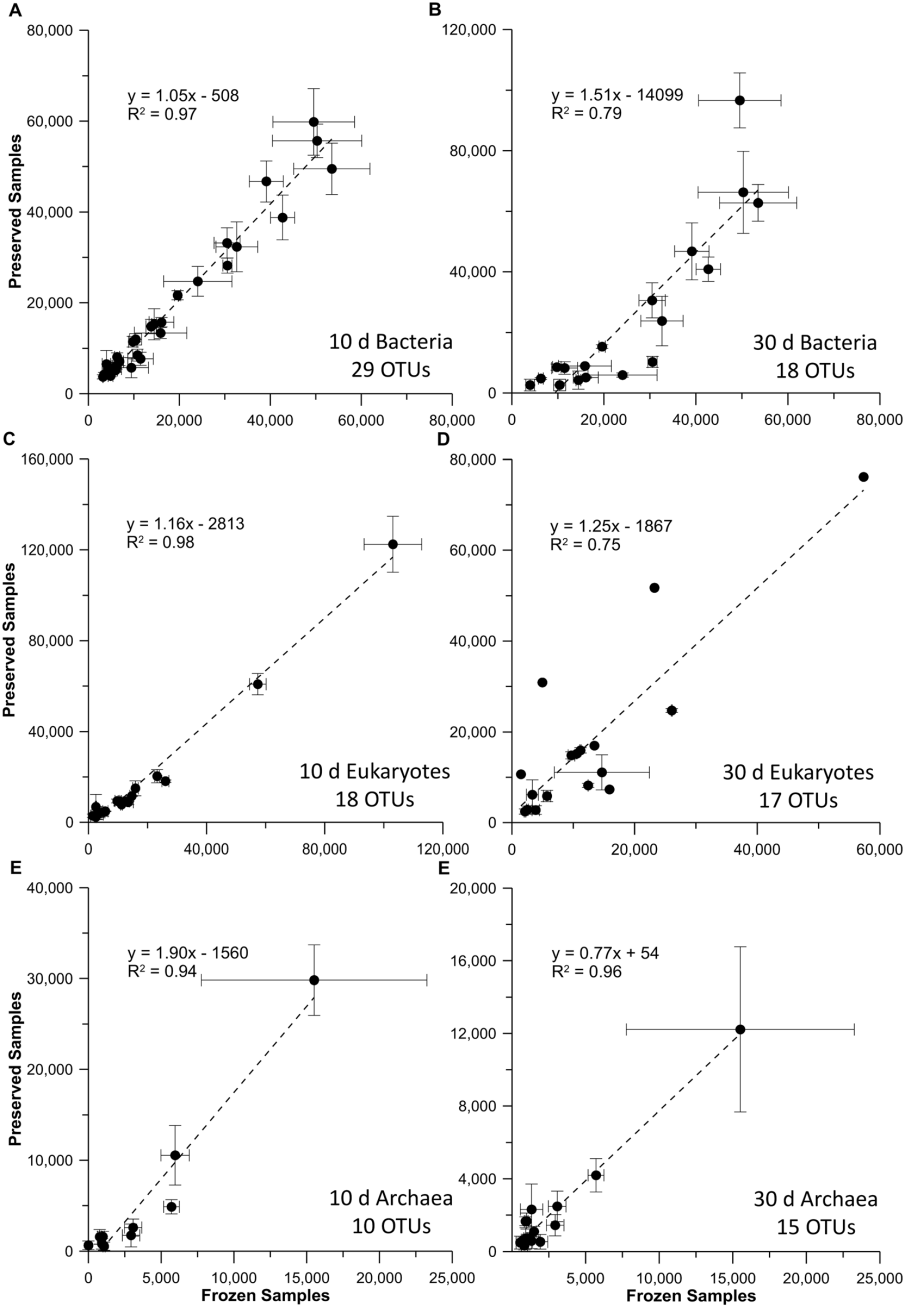
Comparison of RNA-TRFLP peak areas in frozen versus preserved seawater field samples. Results are shown for seawater samples that were frozen, plotted against those preserved with BioDry and stored for either 10 or 30 d for the bacterial (A & B), eukaryotic (C & D), or archaeal (E & F) communities. Values represent averages (n = 3 ± SD) of only those peaks present in both controls and BioDried samples; the number of OTUs used for each analysis is indicated at the bottom-right of each graph.

**Fig 6:**
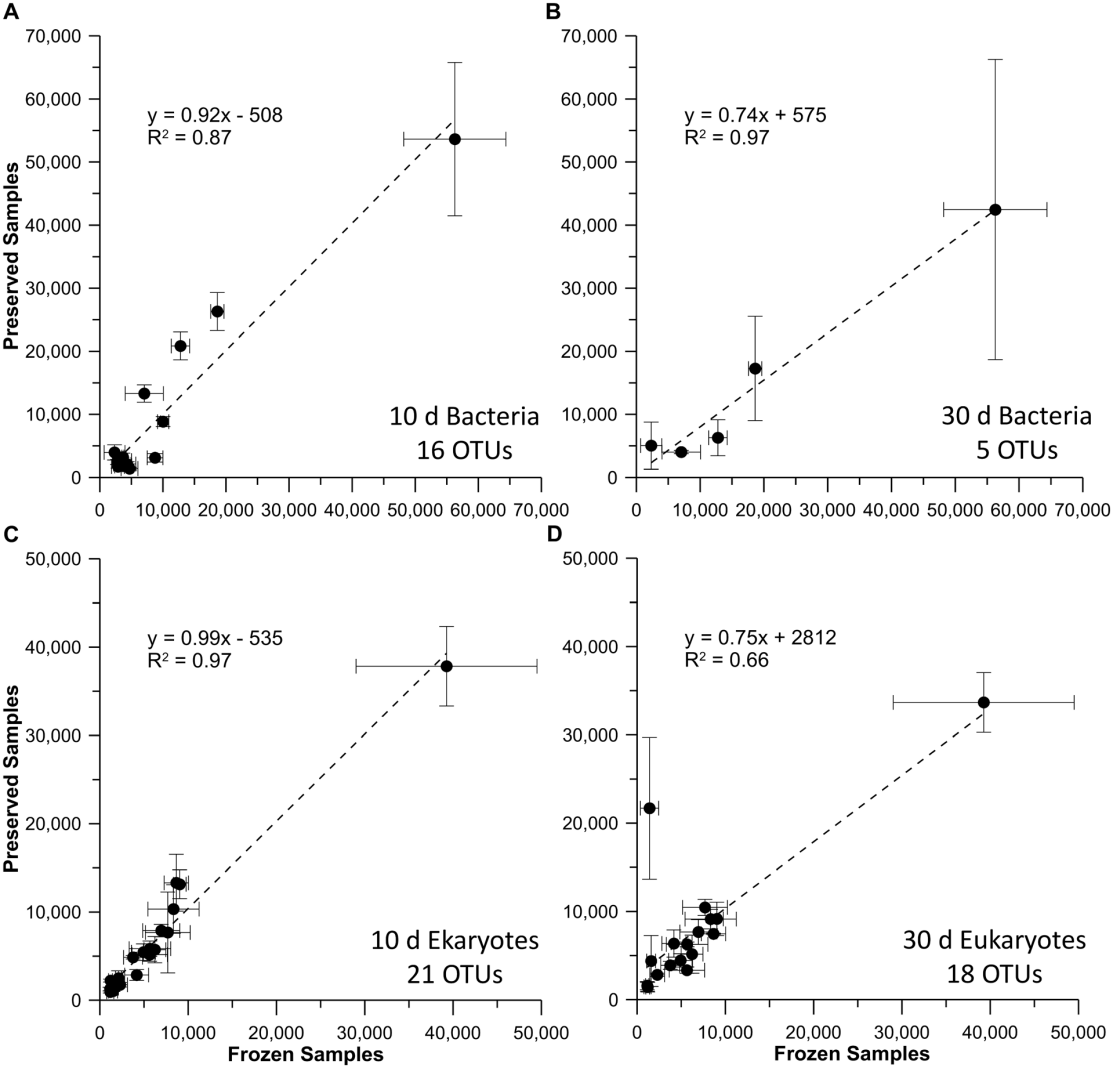
Comparison of RNA-TRFLP peak areas in frozen versus preserved river water field samples. Results are shown for riverine samples that were frozen, plotted against those preserved with BioDry and stored for either 10 or 30 d for the bacterial (A & B) & eukaryotic (C & D) communities. Values represent averages (n = 3 ± SD) of only those peaks present in both controls and BioDried samples; the number of OTUs used for each analysis is indicated at the bottom-right of each graph.

In order to determine if BioDry preservation resulted in selective changes in ribosome abundance in the field samples, the peak areas of those OTUs found in both control and preserved field samples were plotted against each other (T_0_ vs either T_10_ or T_30_), as done for the lab samples above. For the seawater samples, all common OTUs demonstrated a strong linear relationship for the bacterial, eukaryotic, and archaeal profiles (Fig 5). The slopes for the bacterial and eukaryotic T_10_ comparisons (1.05 and 1.16, r^2^ >0.97; Fig 5A and 5C) did not indicate preferential degradation of ribosomal RNAs found in control and preserved samples. However, the T_30_ treatment (Fig 5B and 5D), indicated some differential RNA loss occurred. A similar pattern was seen for the bacterial and eukaryotic profiles from the riverine samples (Fig 6), with a more pronounced decline in common OTUs in the T_30_ comparison, as expected with the decreased nucleic acid yields at that time point. While the correlation between control and BioDry samples was high for archaea in seawater treatments (r^2^ > 0.94), the slope of the relationships varied between 1.90 for the T_10_ and 0.77 for the T_30_ samples (Fig 5E and 5F). Collectively, the data in this report suggest variable abundances and high natural heterogeneity of archaea in these particular samples.

### mRNA and Protein Analysis

**Fig 7:**
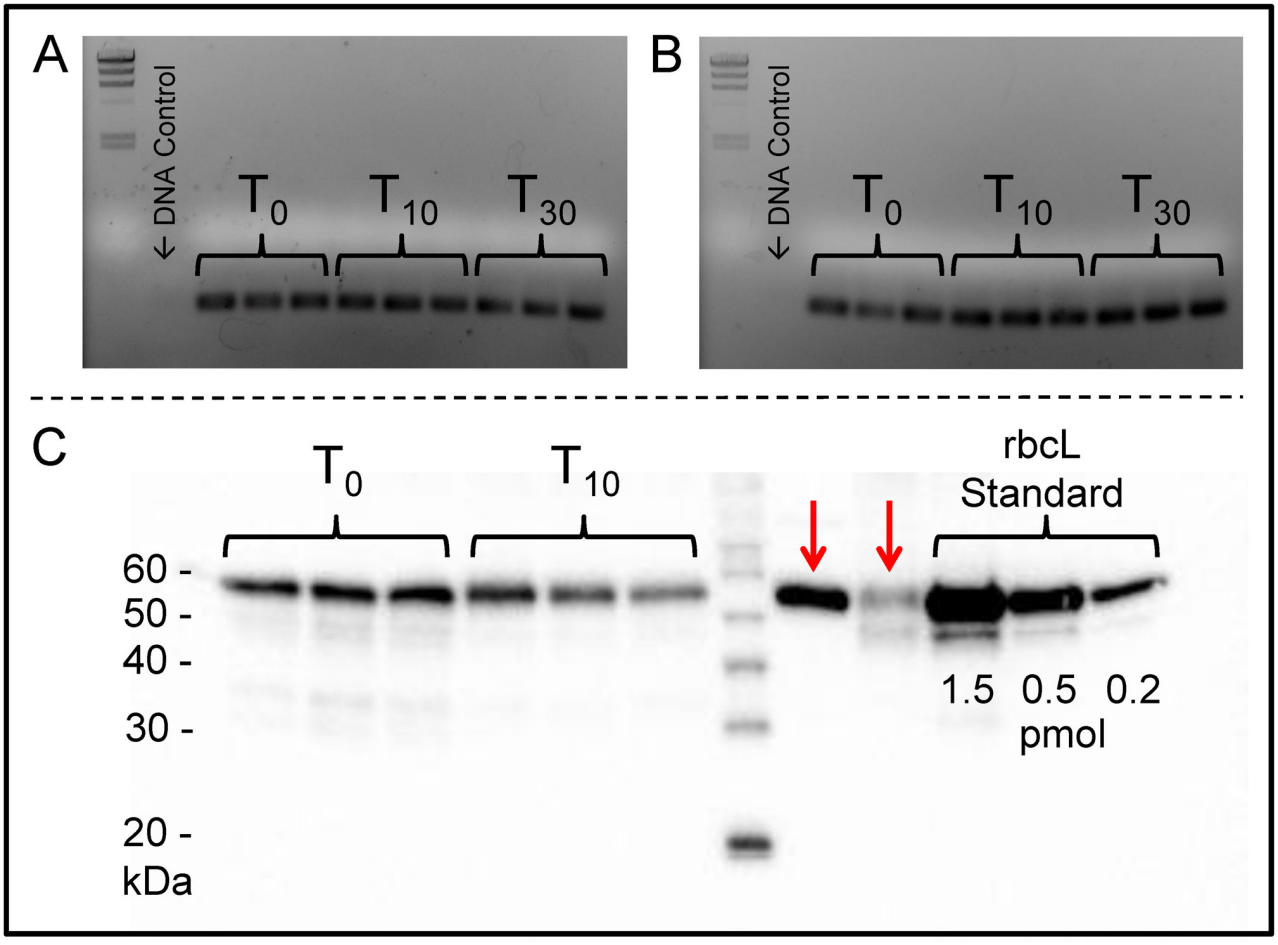
Assessment of protein and *m*RNA integrity for seawater field samples preserved with BioDry. Gene expression for rbcL was assayed using clade-specific primers for diatoms (A) and haptophytes (B). Protein was assayed by western blot using an RbcL antibody (C). Red arrows represent assay positives for *Emiliania huxleyi* CCMP & Glycine max (soy) leaf extract respectively. Frozen controls (T_0_), preserved samples (T_10_ & T_30_ days), as well as the representative DNA/contamination controls are indicated.

Finally, we assessed preservation of *m*RNA and protein through the respective amplification of clade-specific transcripts and immunohybridization with a polyclonal antibody to the large chain component of the photosynthetic protein RuBisCo. Both diatom and haptophyte transcripts in the T_10_ and T_30_ samples amplified with equal strength to those of the T_0_ samples (Fig 7A and 7B), providing evidence that *m*RNA is preserved during BioDry treatment for up to 30 days. All no-RT controls resulted in no amplification indicating any DNA contamination in these extracts was below the PCR detection limits (representatives are indicated in Fig 7A and 7B). Western blot analysis using the RbcL antibody resulted in a clear and strong immunohybridization for triplicate T_10_ preserved samples (Fig 7C), despite a slight, but insignificant decrease in anti-RbcL immunohybridization (Table 1; *t*(4) = 1.48, *p* < 0.05). Importantly, no visible degradation in the protein size was detected (Fig 7C).

## Discussion

Preservation of microbial biomass has often relied on freezing (liquid nitrogen/dry ice-ethanol, -20°C), and/or storage with liquid preservatives (e.g. RNAlater, DMSO-EDTA-salt solutions (DESS), 1–4% paraformaldehyde, 10% Lugol’s, etc.). However, the protection efficacy of macromolecules using these approaches often varies depending on the pre- and post-preservation conditions, the nucleic acid extraction procedures, and the hands of the investigator. For example, Tatangelo et al. report significant differences for soil samples between LifeGuard^™^ for DNA preservation and freezing, DESS, or no-protection by TRFLP analysis of bacterial rRNA genes using the ZR DNA mini prep kit (Zymo Research Corporation, Irvine, CA) [18]. However, these researchers also indicate no significant differences in preserved DNA with water samples using similar methods. In contrast, Grey et al., described comparable preservation of commercial products and DESS for a composite of bacterial cultures using a DNAeasy extraction kit (Qiagen, Valencia, CA) and ARISA analysis (<70% similarity) [7]. While Simister et al. demonstrate large differences in nucleic yields from bacterial communities in preserved sponge tissues using acetone, RNAlater, lyophilization, and liquid nitrogen freezing for 3 different DNA/RNA extraction methods (phenol/chloroform, Trizol, and Qiagen kits) [19]. In contrast, Otteson employed an environmental sampler and RNAlater, and found nearly identical mRNA profiles from microbial communities which were frozen or preserved samples by metatranscriptome pyrosequence analysis using the Mirvana RNA extraction kit (Ambion [4]. Finally, McCarthy et al. 2015 found significant bias in nucleic acid quality and quantity from freshwater microbial communities using no treatment, RNAlater, RNAprotect, RL+lysis buffer for preservation and the Qiagen AllPrep and Mirvana kits or enzymatic extraction methods [20]. The researchers reported 20–40% differences in Bray-Curtis similarities for both the extraction and the preservation methods, with greater variability resulting from the extraction methods.

For this particular study, we were interested in finding a simple storage alternative to use in conjunction with our rapid phenol/chloroform extraction procedure that represented a low-cost, low-power alternative to the tradition freezing/chemical preservation methods and commercial purification kits. Sample desiccation (Biodry) was found to preserve all the major biomolecules of interest for analyzing microbial communities. The BioDry technique requires rapid and nearly complete dehydration of the biomass sample. During optimization, we tested various materials for their ability to dry aquatic biomass (e.g. DrieRite, silica gel, molecular sieve; data not shown). While silica gel and molecular sieve could remove the majority of the moisture from the sample more rapidly than DrieRite (measured as gravimetric loss), only DrieRite reliably preserved the biomass samples. The results from storage under humid conditions (S7 Fig) confirm that samples with residual moisture do not maintain sample integrity over time, and suggests that only DrieRite provided sufficient dehydration for preservation.

We also observed differences in sample preservation for the laboratory vs. the field method. Laboratory tests showed that the BioDry method is effective at preserving DNA and *r*RNA in aquatic microbial samples, while field assays using a portable device showed adequate conservation of *r*RNA, *m*RNA, and proteins, despite apparent loss of DNA with time. Comparison of the results from the methods verification and temperature/humidity tests (Fig 1 & S7 Fig) suggest that the difference in DNA conservation is related to the ability of the BioDry apparatus to control for environmental conditions (e.g. humidity). For example, the laboratory samples were processed in a building with relatively cool, dry air using a recirculating desiccation system (S1 Fig), resulting in reliable preservation of all samples for up to 30 days (Figs 1–3). Conversely, both sets of environmental field samples were collected outside on warm days with high humidity. Furthermore, the portable system was not configured to recirculate the dry air, rather ambient air was forced over the desiccant cartridge and expelled over the filter for drying (S4 and S5 Figs). The result was a less efficient preservation of samples (Fig 4). Both the high and low (90 vs. 50 SCFH) air flow treatments in the lab tests demonstrated comparable preservation (Fig 1), indicates that the lower air-flow rate of the portable device (50 SCFH) was not a major factor contributing to the DNA loss in the field tests.

A reduction of the drying time to 8 min for the field samples was another potential cause for insufficient preservation when using the portable device. Initially, this was deemed an acceptable reduction in drying time given that there was no observable difference between 8, 10, and 20 m drying using the laboratory equipment, and was done as an attempt to conserve battery life. Higher drying times may be necessary to achieve optimal preservation with an, “open” system, or possibly a portable device will only be as effective as the laboratory apparatus if it is configured to recirculate the desiccated air as well. However, the correlation coefficients of the peak area relationships for the T_0_:T_30_ samples (Figs 5 and 6) suggest, that even with some loss of biomass when using the portable BioDry device, the dominant organisms in the community were largely conserved in their original relative proportions. Similar findings have been reported by McCarthy et al. [20]. It is anticipated that with the optimization of the field unit, sample conservation similar to that of the lab tests can be achieved and no diversity will be lost.

In conclusion, BioDry is effective at preserving the key biomolecules (DNA, RNAs, and proteins) in single aquatic microbial samples with the integrity necessary for molecular analysis. This includes identification of not only the resident community, but also the active individuals via functional gene expression and catalytic proteins, collectively linking metabolic and biogeochemical activity. Furthermore, preserved samples can be stored at room temperature for extended periods (up to 30 d), indicating that biodegradative enzymatic activities (e.g. nucleases, proteases, etc.) are halted by the Biodry process. This finding is in contrast with preservation of macromolecules using the most widely used liquid preservative, RNALater [4,7,9]. For example, cold storage is necessary to preserve DNA for periods > 1 week with RNALater [7] (see also manufacturer’s protocol), and has also been suggested for ideal results when preserving the proteome as well [9]. Likewise, it has still been recommended that flash freezing be employed whenever possible when preserving RNA molecules [17]. Lastly, liquid preservatives are often expensive, and require ≥ 5:1 volume of preservative to sample ratios to be effective. Conversely, once the portable system is optimized, BioDry will require only rechargeable batteries and desiccant.

Given BioDry successfully preserves *m*RNA, the most labile of cellular macromolecules, the procedure is likely effective at preserving other biomolecules of interest, such as lipids, metabolites and carbohydrates. Having a reliable means of preserving aquatic biomass in environmental samples at room temperature has the potential to greatly increase our understanding of microbial ecology given that sampling often needs to be done in remote areas where power, refrigeration/freezer equipment, and complicated shipping procedures are not readily available [17]. Finally, our findings indicate that, with optimization, a portable field version of the BioDry method will attain the level of preservation observed when using the laboratory device. This could then be adapted for autonomous platforms to improve spatial and temporal resolution of biomass sampling and significantly improve our understanding of the factors controlling microbial populations in the ocean and almost any other environment.

## Supporting Information

S1 Fig AC powered vacuum pump adapted to BioDry filtered aquatic biomass samples. (TIF)

S2 Fig Image of sample being collected onto a filter. Aquatic biomass can be collected using either a swinnex (A) or a syringe filter (B) while using either a syringe (A) or portable compressor (B).
(TIF)

S3 Fig Pre-prepared filter holders. A centrifuge tube is cut off at the bottom and a section of Tygon tubing is attached (A). The newly filtered sample is placed inside the tube (B), and then connected to another cut-off tube via a short length of tube (C). This can then be fixed at either end to the in-lab apparatus (see S1 Fig), or to the desiccation syringe of the portable apparatus (D). After desiccation is complete the caps are closed at either end for storage (C).
(TIF)

S4 Fig Image and schematic of portable BioDry apparatus. The portable apparatus consisted of a Ryobi compressor designed to filter and preserve aquatic samples. Plumbing was adapted to be successively diverted to first a sample syringe to load the sample onto a filter, then to a desiccant syringe to preserve the sample.
(TIF)

S5 Fig Cleaving of syringe filters (A), followed by attachment of filter to DrieRite syringe (B). (TIF)

S6 Fig Agarose gels from BioDry quality control tests. Panel A—Procedural control tests: Showing total nucleic acid extracts from filters that were either immediately frozen (frozen controls), or subjected to the BioDry procedure, then flash frozen (procedural control). Panel B—Sample integrity and procedural contamination tests: Showing PCR results from extracts of “Air-Blank” filters which resulted in no amplification (left 2 lanes), while seawater samples resulted in strong amplification (right 2 lanes). Results shown for both types of QC tests are representative of several such tests.
(TIF)

S7 Fig Agarose gel of DNA/RNA extracts showing the effects of temperature and humidity on laboratory samples preserved with BioDry. Extracts of the controls are shown with preserved samples that were stored at 18, 27, and 37°C in either dry or humid conditions respectively.
(TIF)

S8 Fig Bacterial DNA-TRFLP profiles from the laboratory-based aquarium samples. Showing the control profiles (A; n = 2), as well as those preserved and stored for 15 days (B & C; n = 4 each) or for 30 days (D & E; n = 4 each). Those BioDried under high flow-rates are represented by profiles B & D, and low flow-rates by C & E. Each BioDried profile (B-E), displays both replicates dried for 10 min (blue & black profiles) and those dried for 20 min (red & orange profiles) for their respective flow-rates and storage times.
(TIF)

S9 Fig Bacterial RNA-TRFLP profiles from the laboratory-base aquarium samples. Showing the control profiles (A; n = 2), as well as those preserved and stored for 15 days (B & C; n = 4 each) or for 30 days (D & E; n = 4 each). Those BioDried under high flow-rates are represented by profiles B & D, and low flow-rates by C & E. Each BioDried profile (B-E), displays both replicates dried for 10 min (blue & black profiles) and those dried for 20 min (red & orange profiles) for their respective flow-rates and storage times.
(TIF)

S10 Fig RT-TRFLP profiles for the bacterial community from the seawater field samples. Panels display the T_0_ control samples (A) as well as those preserved for T_10_ and T_30_ days (B & C) respectively (n = 3 for each). The black arrows indicate the gain of a significant peak in multiple replicates within a treatment.
(TIF)

S11 Fig RT-TRFLP profiles for the eukaryotic community from the seawater field samples. Panels display the T_0_ control samples (A) as well as those preserved for T_10_ and T_30_ days (B & C) respectively (n = 3 for each). The red arrows indicate the loss of a significant peak in multiple replicates within a treatment.
(TIF)

S12 Fig RT-TRFLP profiles for the archaeal community from the seawater field samples. Panels display the T_0_ control samples (A) as well as those preserved for T_10_ and T_30_ days (B & C) respectively (n = 3 for each).
(TIF)

S13 Fig RT-TRFLP profiles for the bacterial community from the river water field samples. Panels display the T_0_ control samples (A) as well as those preserved for T_10_ and T_30_ days (B & C) respectively (n = 3, control; n = 2 for 10 & 30d).
(TIF)

S14 Fig RT-TRFLP profiles for the eukaryotic community from the river water field samples. Panels display the T_0_ control samples (A) as well as those preserved for T_10_ and T_30_ days (B & C) respectively (n = 3 for each).
(TIF)

S1 Table Sequence Read Archive project, sample, experiment, and run accession numbers for 454 sequence libraries from the method verification samples—NCBI 454-pyrosequence accession numbers for the T_0_ control and BioDried T_15_ and T_30_ samples. (PDF)

S2 Table Closest match species taxonomic ID for 454 sequences from the seawater samples– 454-pyrosequence species identification (closest match, percent similarity) and relative percent contribution of organisms detected in the T_0_ control and BioDried T_15_ and T_30_ samples. (PDF)

S3 Table Bray-Curtis similarity index of the DNA 454-pyrosequence analysis comparing the bacterial community structures of the T_0_, T_15_, T_30_. (PDF)

S4 Table Bray-Curtis similarity index of the DNA-TRFLP analysis comparing the bacterial community structures of all T_0_, T_15_, and T_30_ replicates from the method verification tests. (PDF)

S5 Table Bray-Curtis similarity index of the RNA-TRFLP analysis comparing the bacterial community structures of all T_0_, T_15_, and T_30_ replicates from the method verification tests. (PDF)

S6 Table Bray-Curtis similarity index of the RNA-TRFLP analysis comparing the seawater bacterial community structures of all T_0_, T_10_, and T_30_ replicates from the field tests. (PDF)

S7 Table Bray-Curtis similarity index of the RNA-TRFLP analysis comparing the seawater eukaryotic community structures of all T_0_, T_10_, and T_30_ replicates from the field tests. (PDF)

S8 Table Bray-Curtis similarity index of the RNA-TRFLP analysis comparing the river bacterial community structures of all T_0_, T_10_, and T_30_ replicates from the field tests. (PDF)

S9 Table Bray-Curtis similarity index of the RNA-TRFLP analysis comparing the river eukaryotic community structures of all T_0_, T_10_, and T_30_ replicates from the field tests. (PDF)

S10 Table Bray-Curtis similarity index of the RNA-TRFLP analysis comparing the seawater archaeal community structures of all T_0_, T_10_, and T_30_ replicates from the field tests. (PDF)
