# Supplementary figures and images for: BioDry: An Inexpensive, Low-Power Method to Preserve Aquatic Microbial Biomass at Room Temperature
Source: PLoS One. 2015 Dec 28;10(12):e0144686. doi: 10.1371/journal.pone.0144686 (PMC4692454; doi:10.1371/journal.pone.0144686)

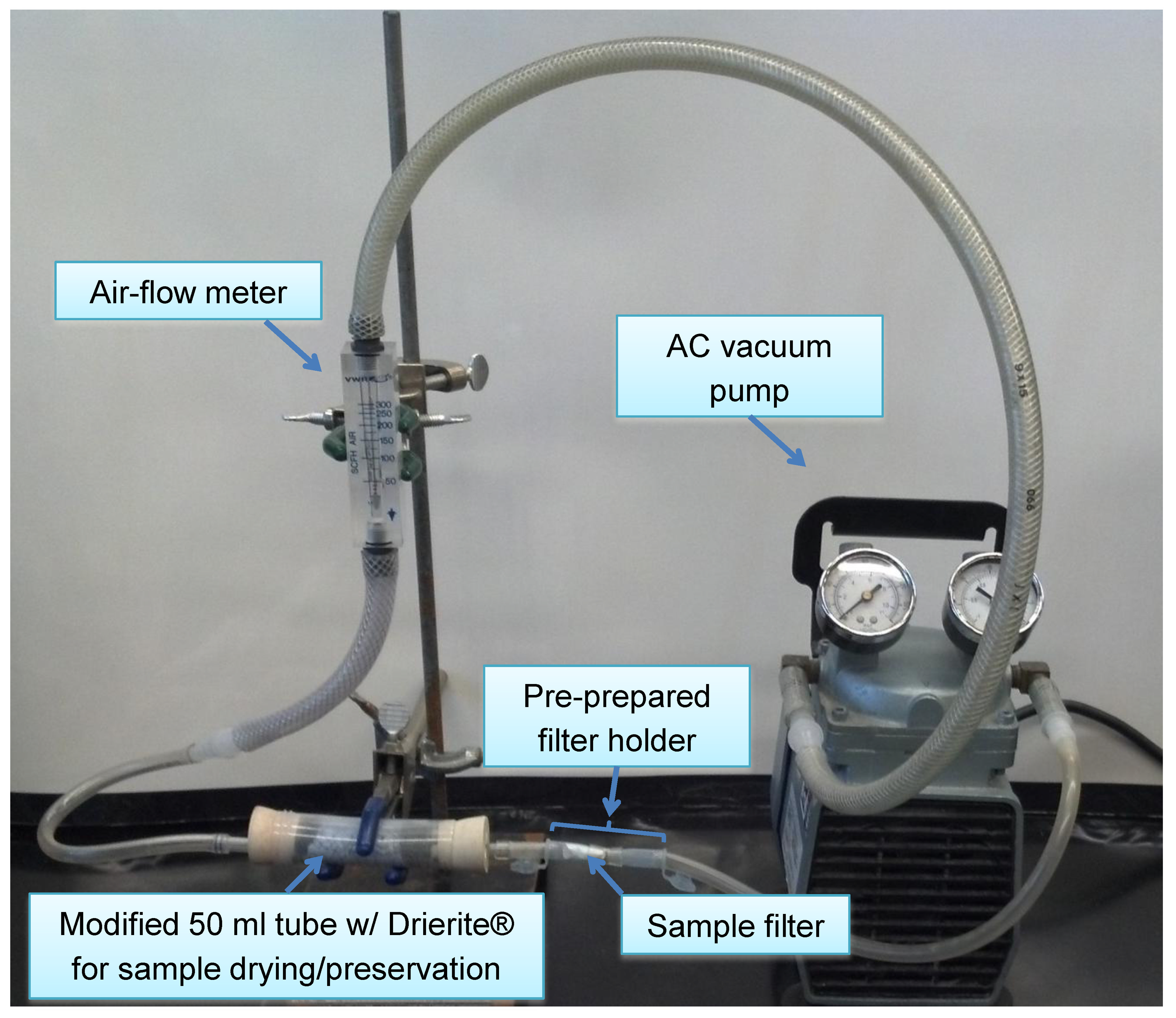

Supplement: S1 Fig — (TIF) [file pone.0144686.s001.tif]

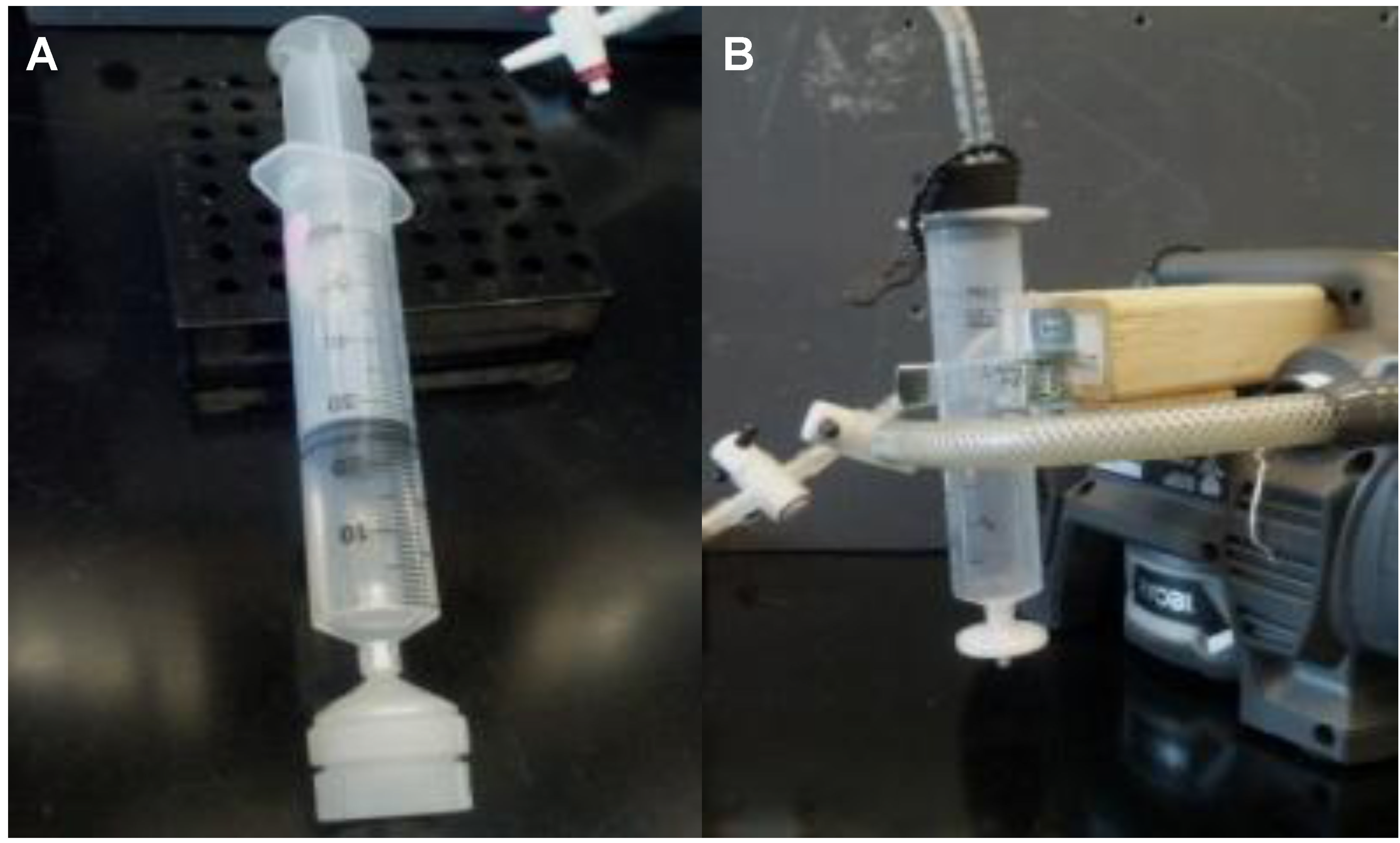

Supplement: S2 Fig — Aquatic biomass can be collected using either a swinnex (A) or a syringe filter (B) while using either a syringe (A) or portable compressor (B). (TIF) [file pone.0144686.s002.tif]

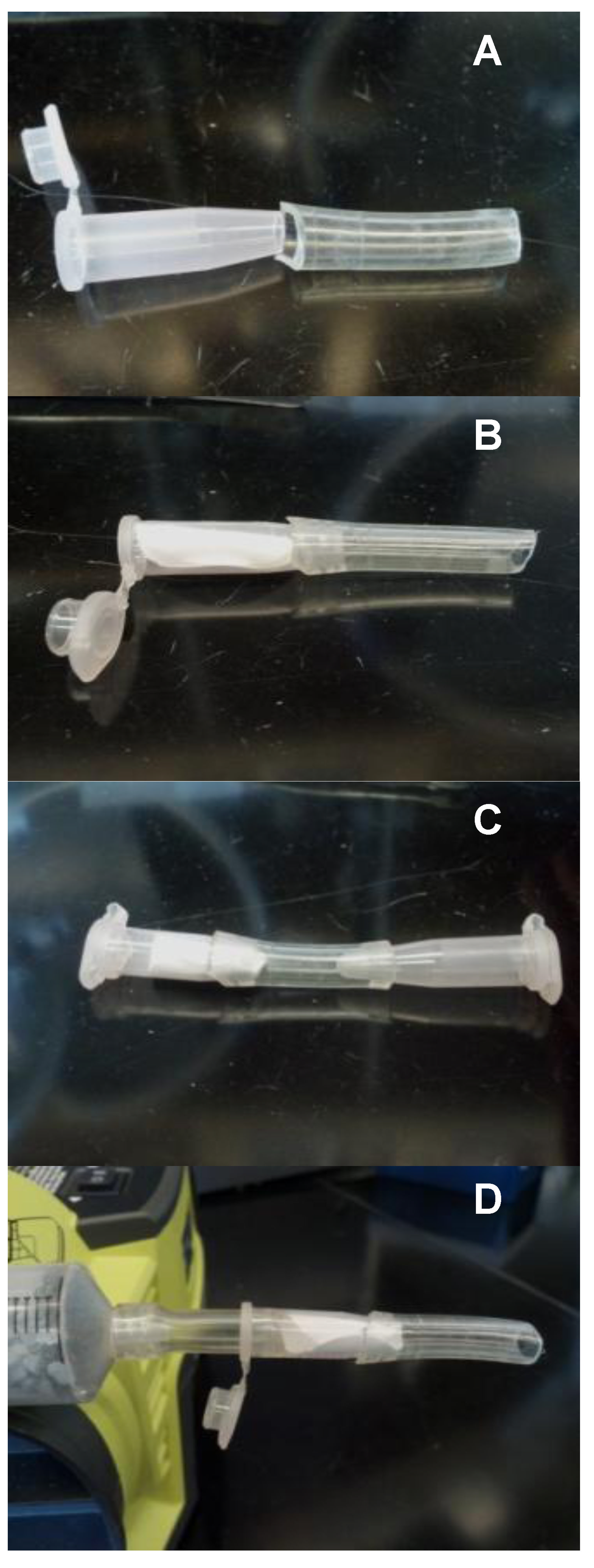

Supplement: S3 Fig — A centrifuge tube is cut off at the bottom and a section of Tygon tubing is attached (A). The newly filtered sample is placed inside the tube (B), and then connected to another cut-off tube via a short length of tube (C). This can then be fixed at either end to the in-lab apparatus (see S1 Fig), or to the desiccation syringe of the portable apparatus (D). After desiccation is complete the caps are closed at either end for storage (C). (TIF) [file pone.0144686.s003.tif]

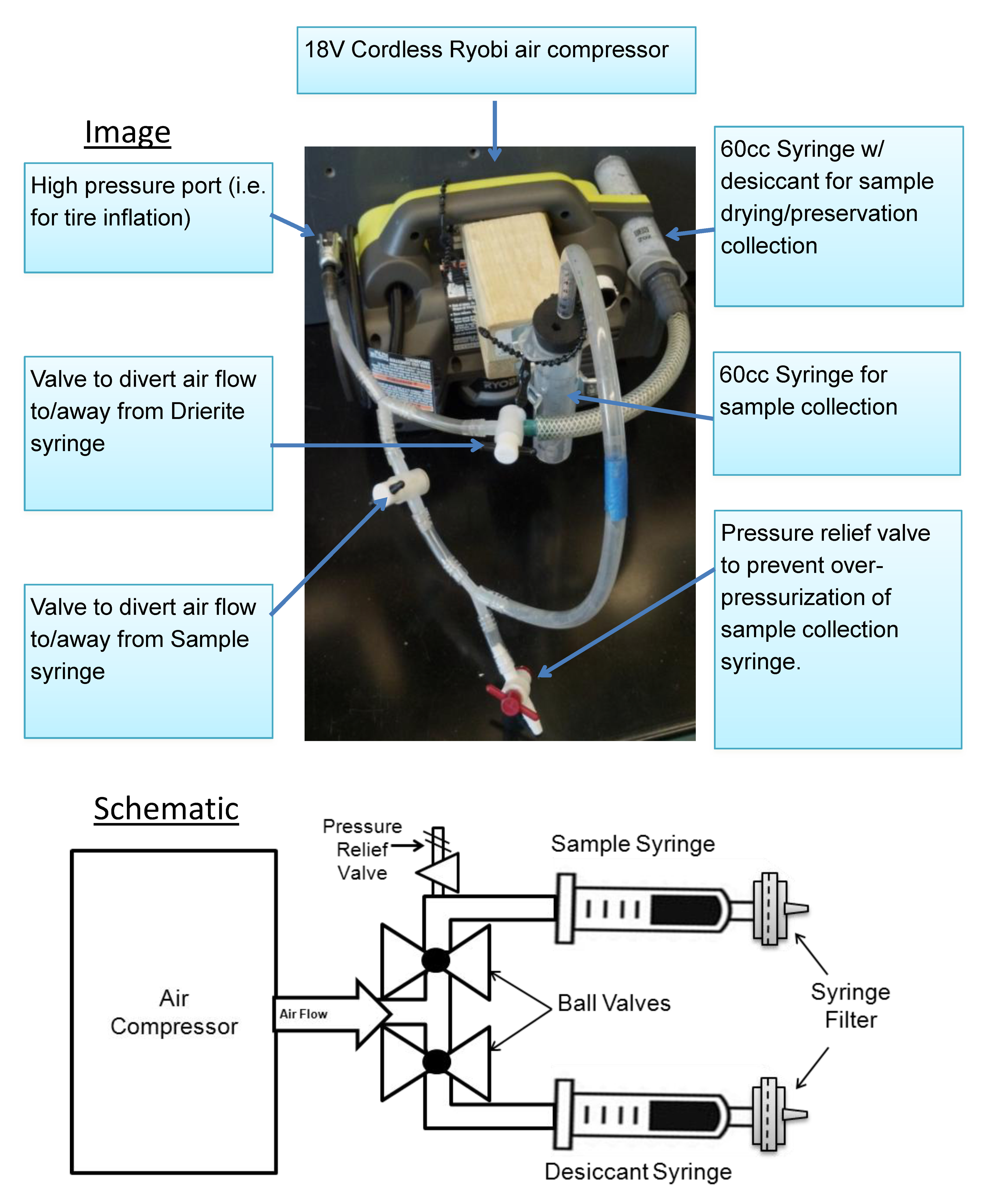

Supplement: S4 Fig — The portable apparatus consisted of a Ryobi compressor designed to filter and preserve aquatic samples. Plumbing was adapted to be successively diverted to first a sample syringe to load the sample onto a filter, then to a desiccant syringe to preserve the sample. (TIF) [file pone.0144686.s004.tif]

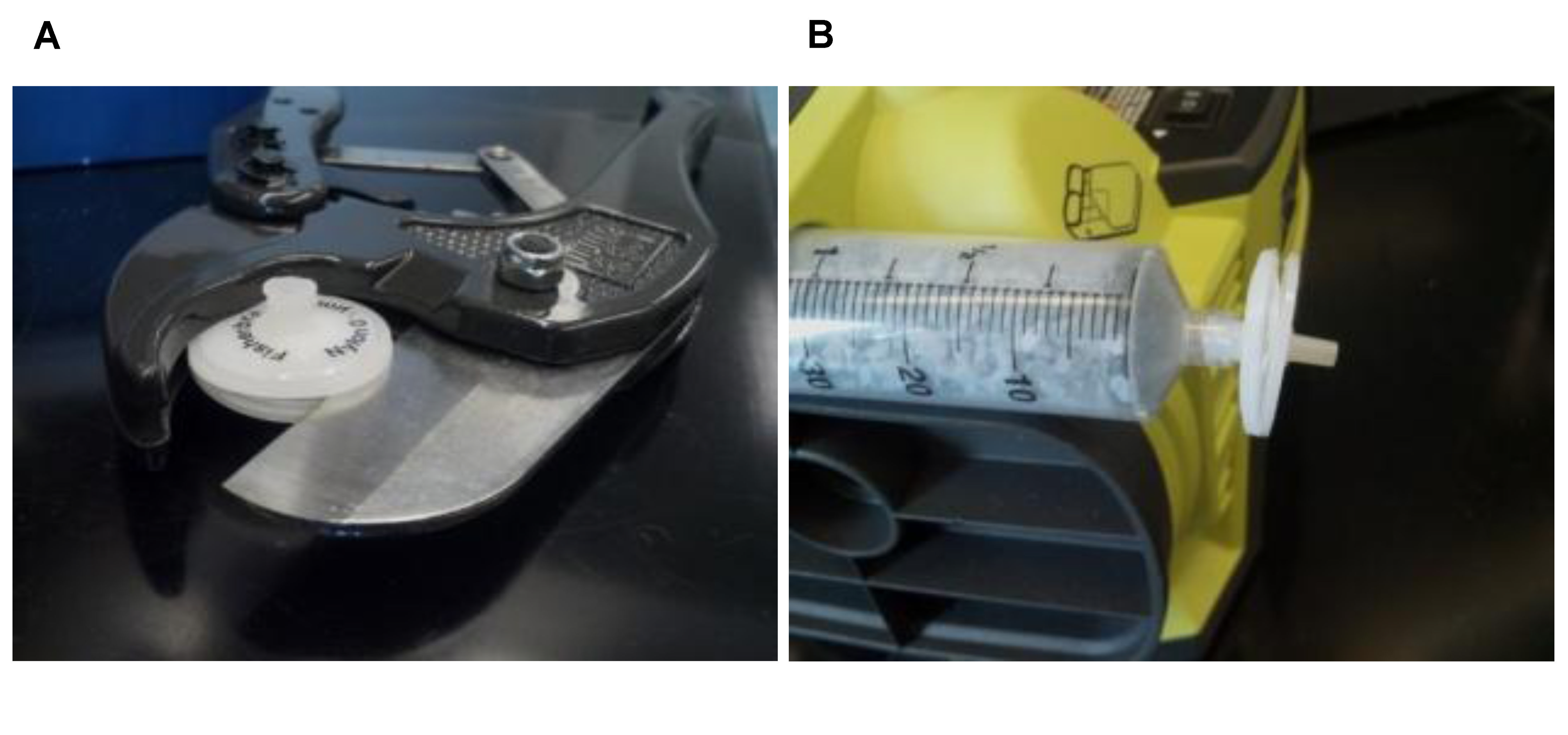

Supplement: S5 Fig — (TIF) [file pone.0144686.s005.tif]

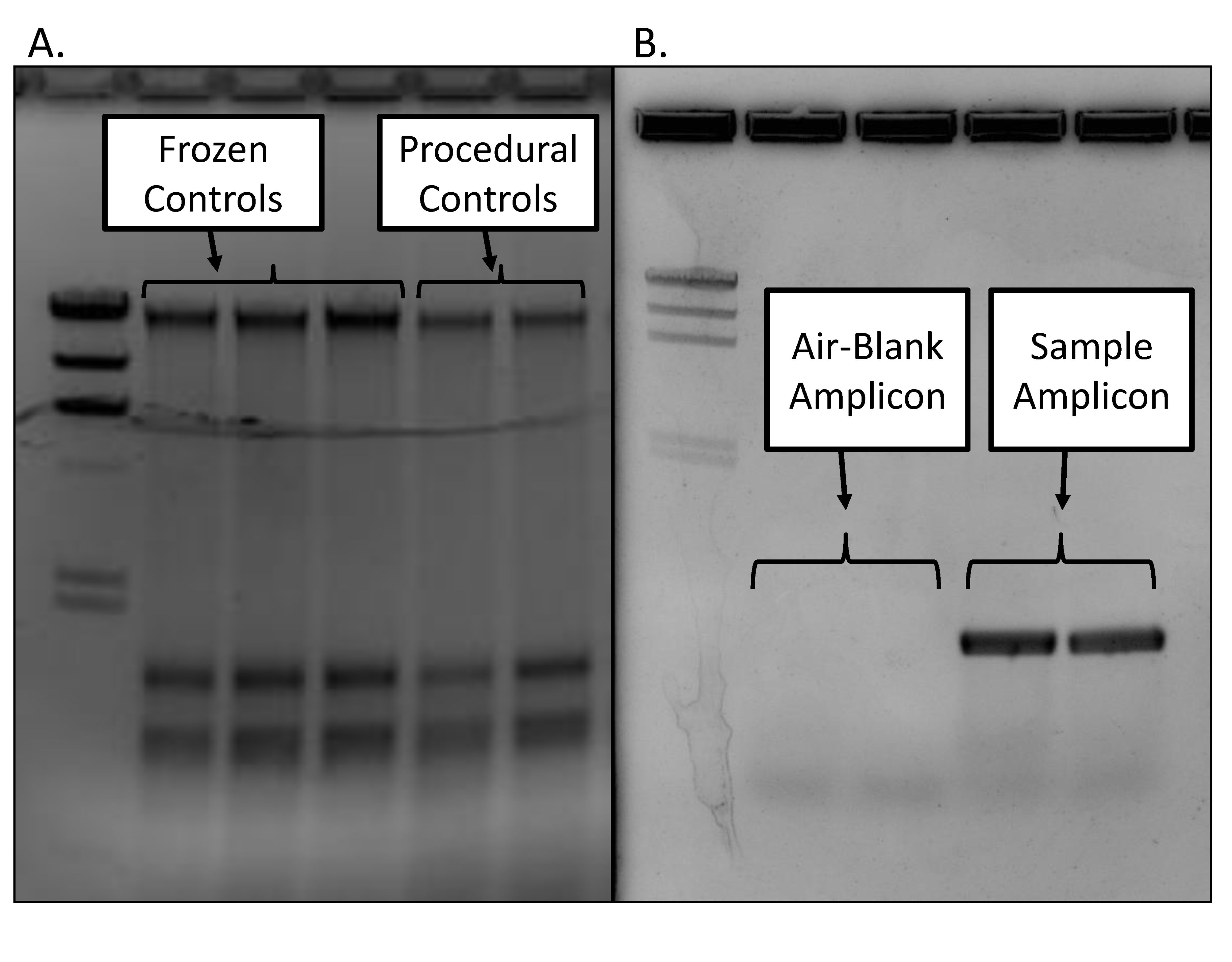

Supplement: S6 Fig — Panel A—Procedural control tests: Showing total nucleic acid extracts from filters that were either immediately frozen (frozen controls), or subjected to the BioDry procedure, then flash frozen (procedural control). Panel B—Sample integrity and procedural contamination tests: Showing PCR results from extracts of “Air-Blank” filters which resulted in no amplification (left 2 lanes), while seawater samples resulted in strong amplification (right 2 lanes). Results shown for both types of QC tests are representative of several such tests. (TIF) [file pone.0144686.s006.tif]

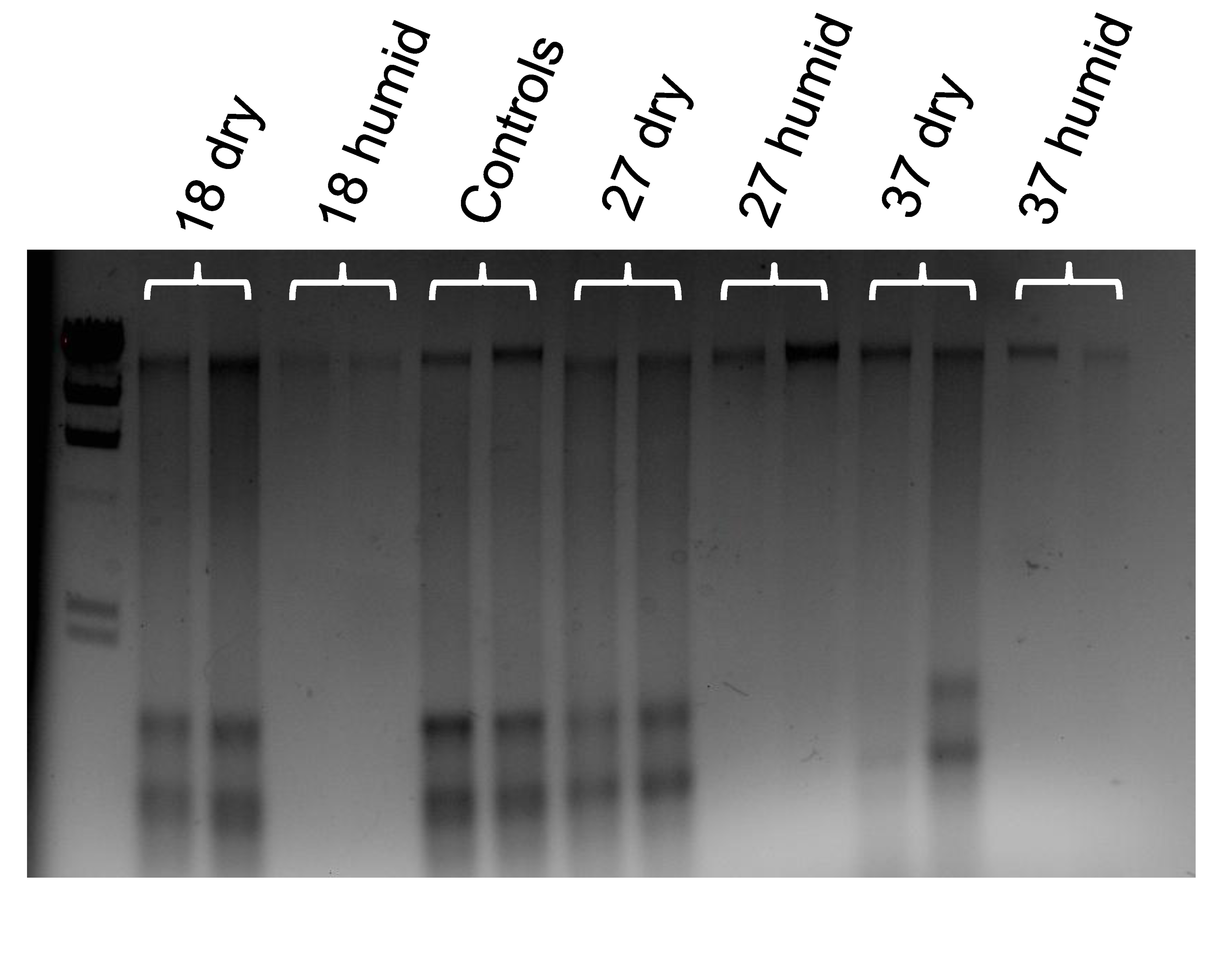

Supplement: S7 Fig — Extracts of the controls are shown with preserved samples that were stored at 18, 27, and 37°C in either dry or humid conditions respectively. (TIF) [file pone.0144686.s007.tif]

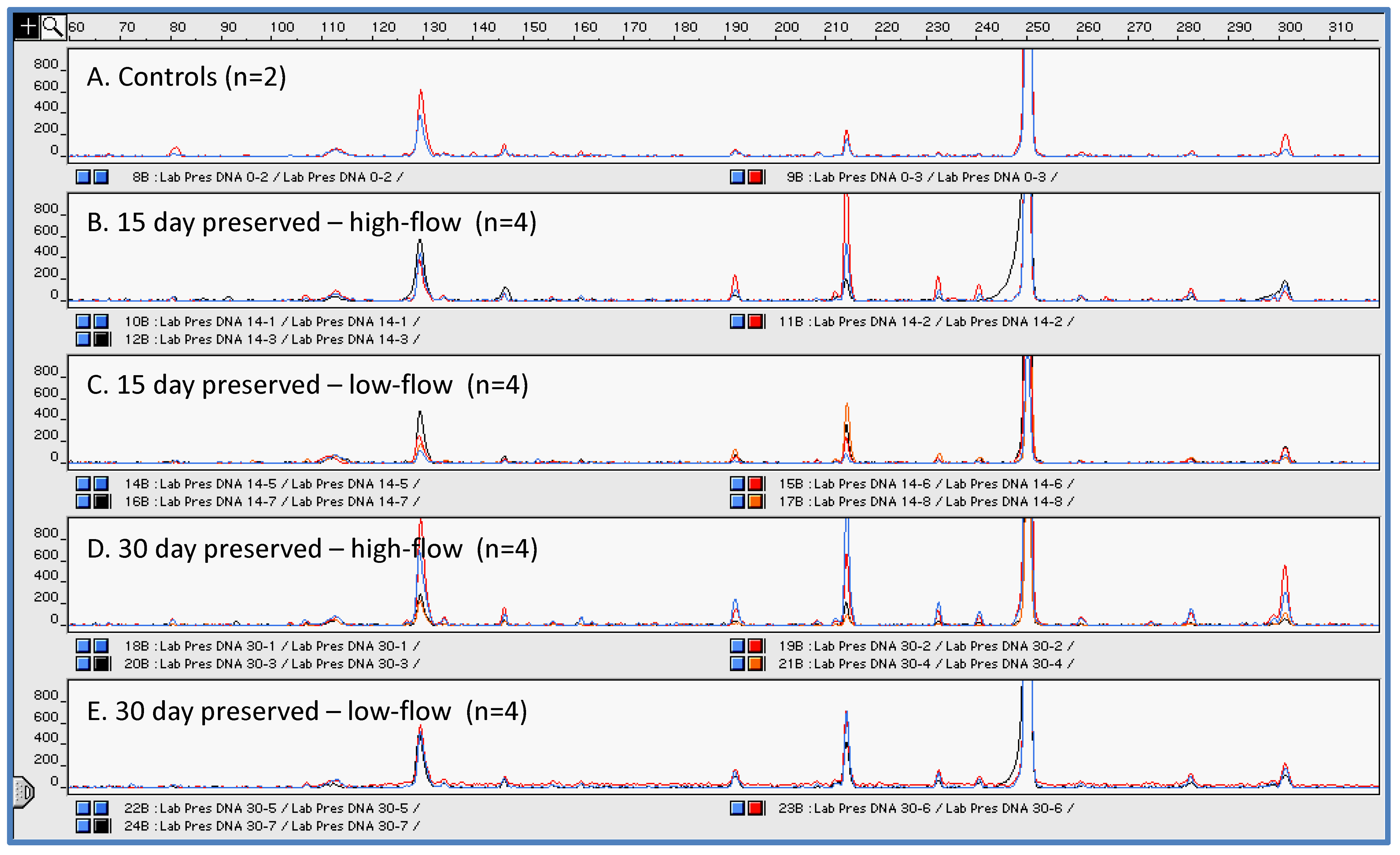

Supplement: S8 Fig — Showing the control profiles (A; n = 2), as well as those preserved and stored for 15 days (B & C; n = 4 each) or for 30 days (D & E; n = 4 each). Those BioDried under high flow-rates are represented by profiles B & D, and low flow-rates by C & E. Each BioDried profile (B-E), displays both replicates dried for 10 min (blue & black profiles) and those dried for 20 min (red & orange profiles) for their respective flow-rates and storage times. (TIF) [file pone.0144686.s008.tif]

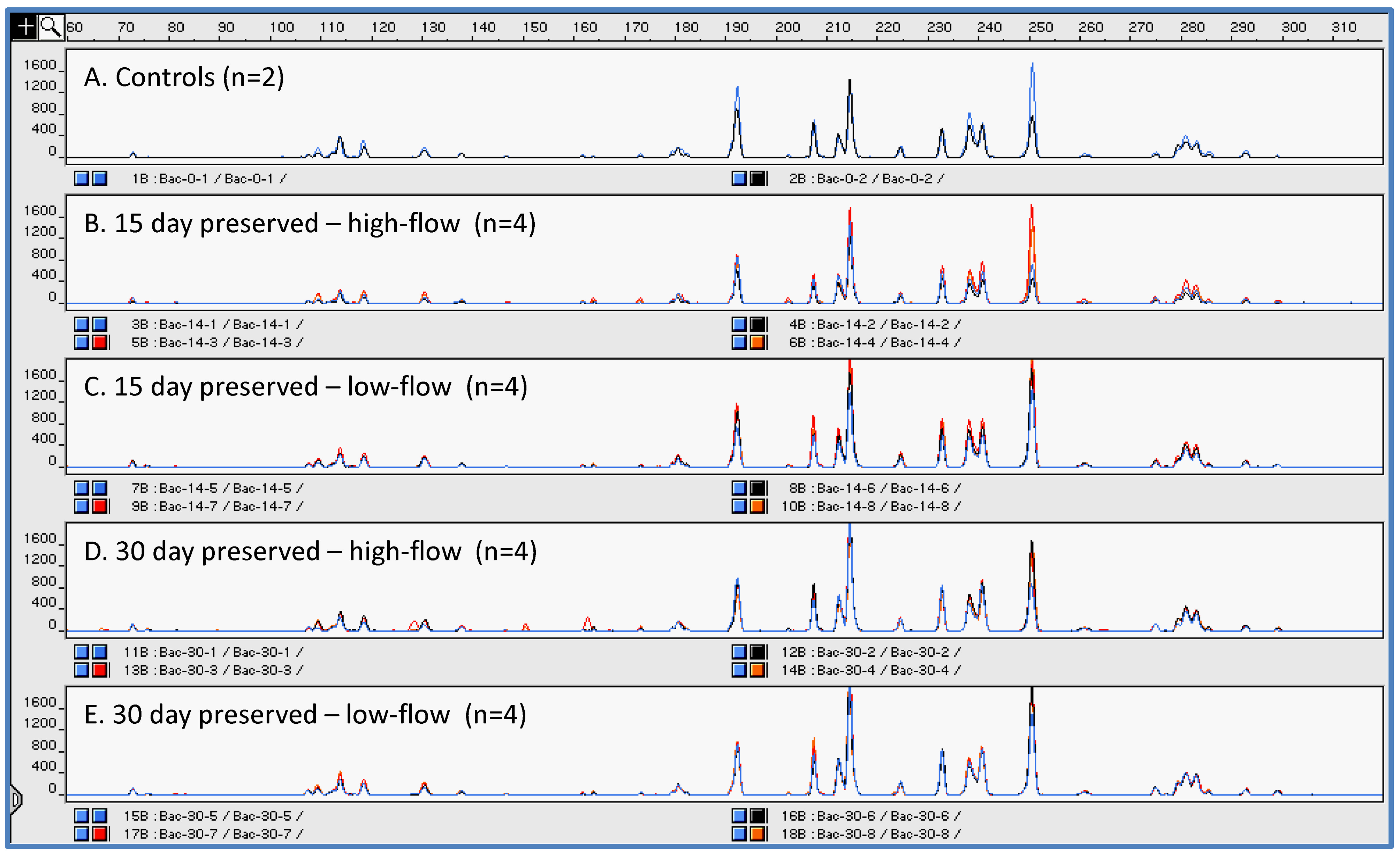

Supplement: S9 Fig — Showing the control profiles (A; n = 2), as well as those preserved and stored for 15 days (B & C; n = 4 each) or for 30 days (D & E; n = 4 each). Those BioDried under high flow-rates are represented by profiles B & D, and low flow-rates by C & E. Each BioDried profile (B-E), displays both replicates dried for 10 min (blue & black profiles) and those dried for 20 min (red & orange profiles) for their respective flow-rates and storage times. (TIF) [file pone.0144686.s009.tif]

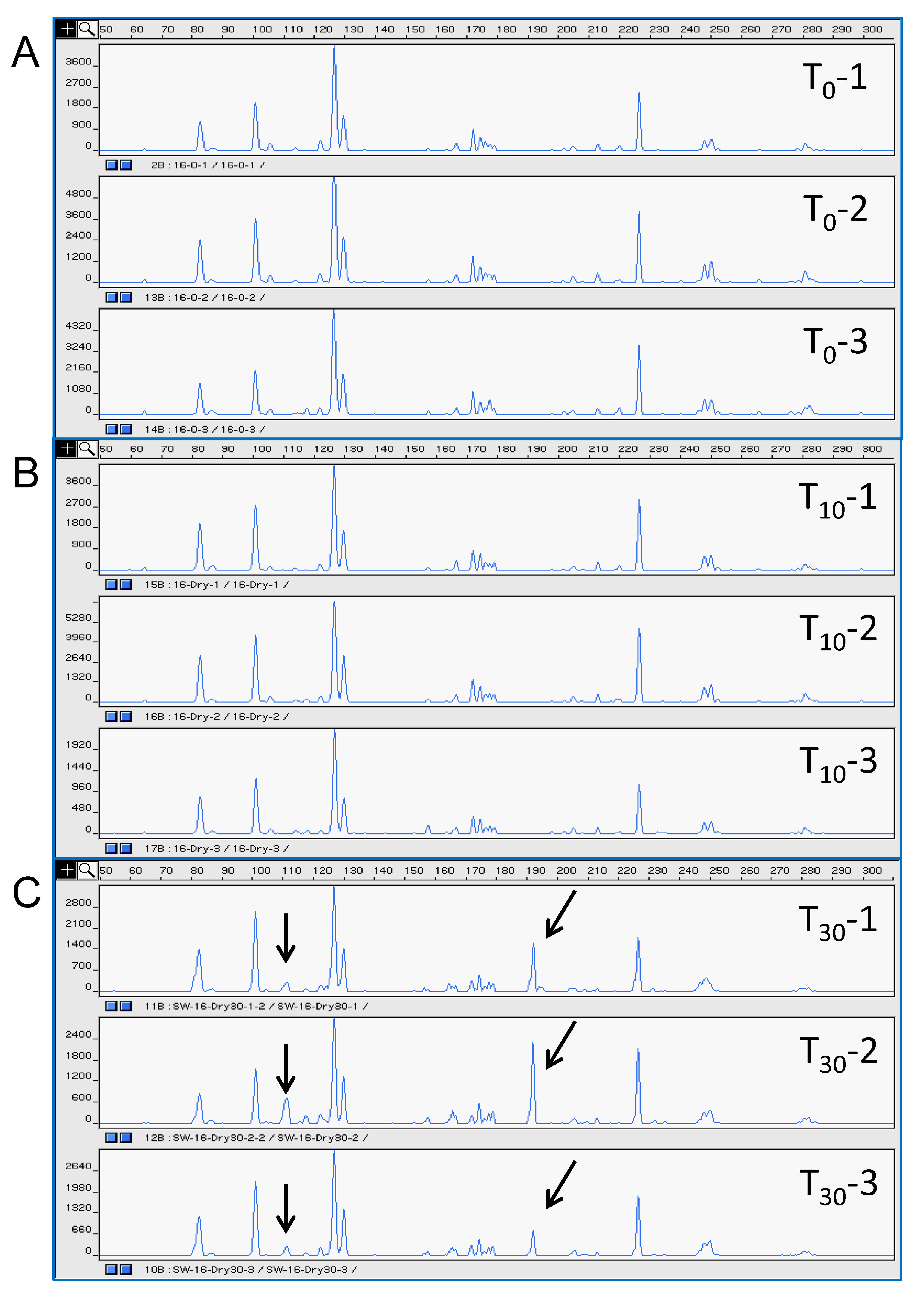

Supplement: S10 Fig — Panels display the T0 control samples (A) as well as those preserved for T10 and T30 days (B & C) respectively (n = 3 for each). The black arrows indicate the gain of a significant peak in multiple replicates within a treatment. (TIF) [file pone.0144686.s010.tif]

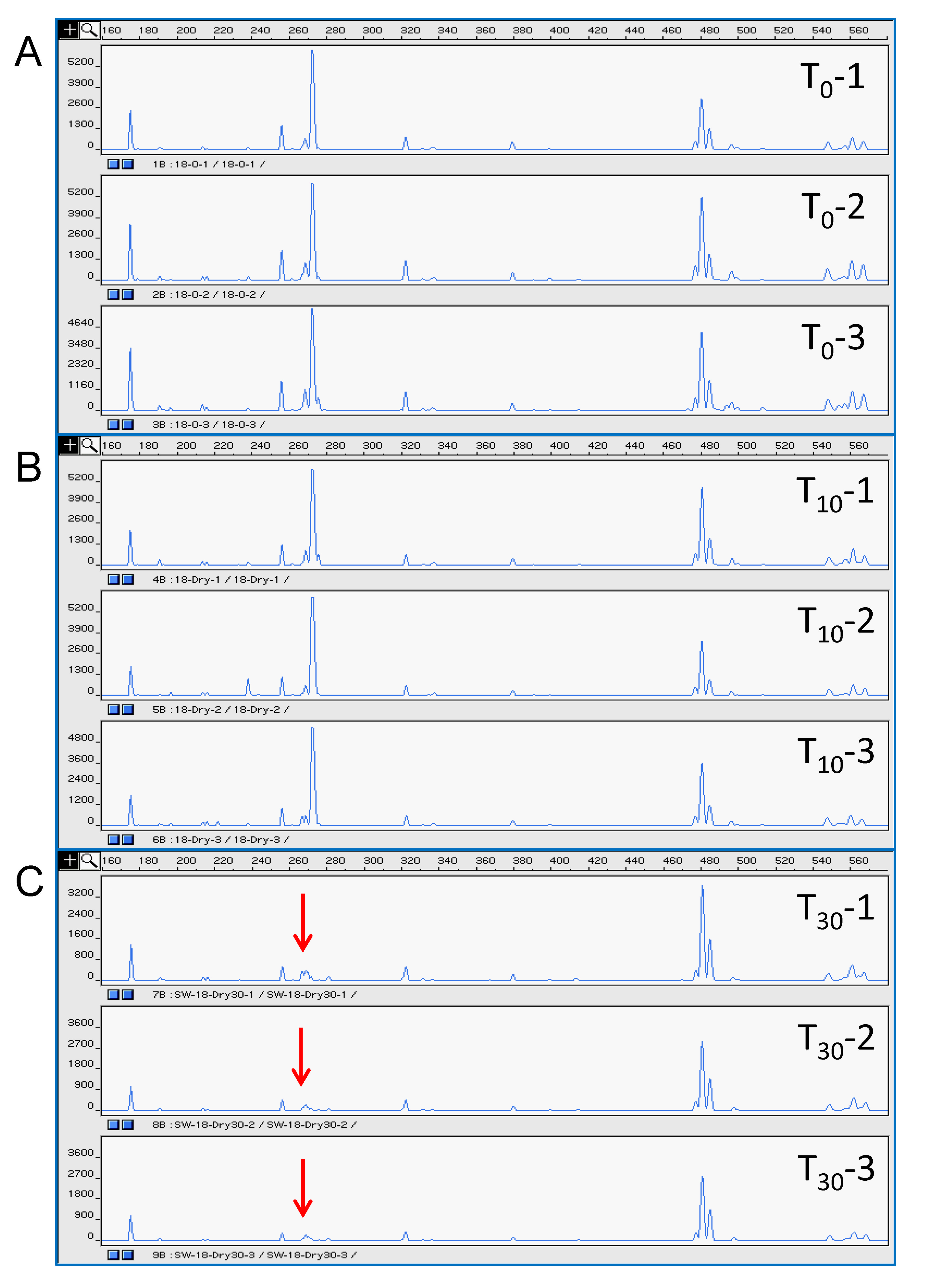

Supplement: S11 Fig — Panels display the T0 control samples (A) as well as those preserved for T10 and T30 days (B & C) respectively (n = 3 for each). The red arrows indicate the loss of a significant peak in multiple replicates within a treatment. (TIF) [file pone.0144686.s011.tif]

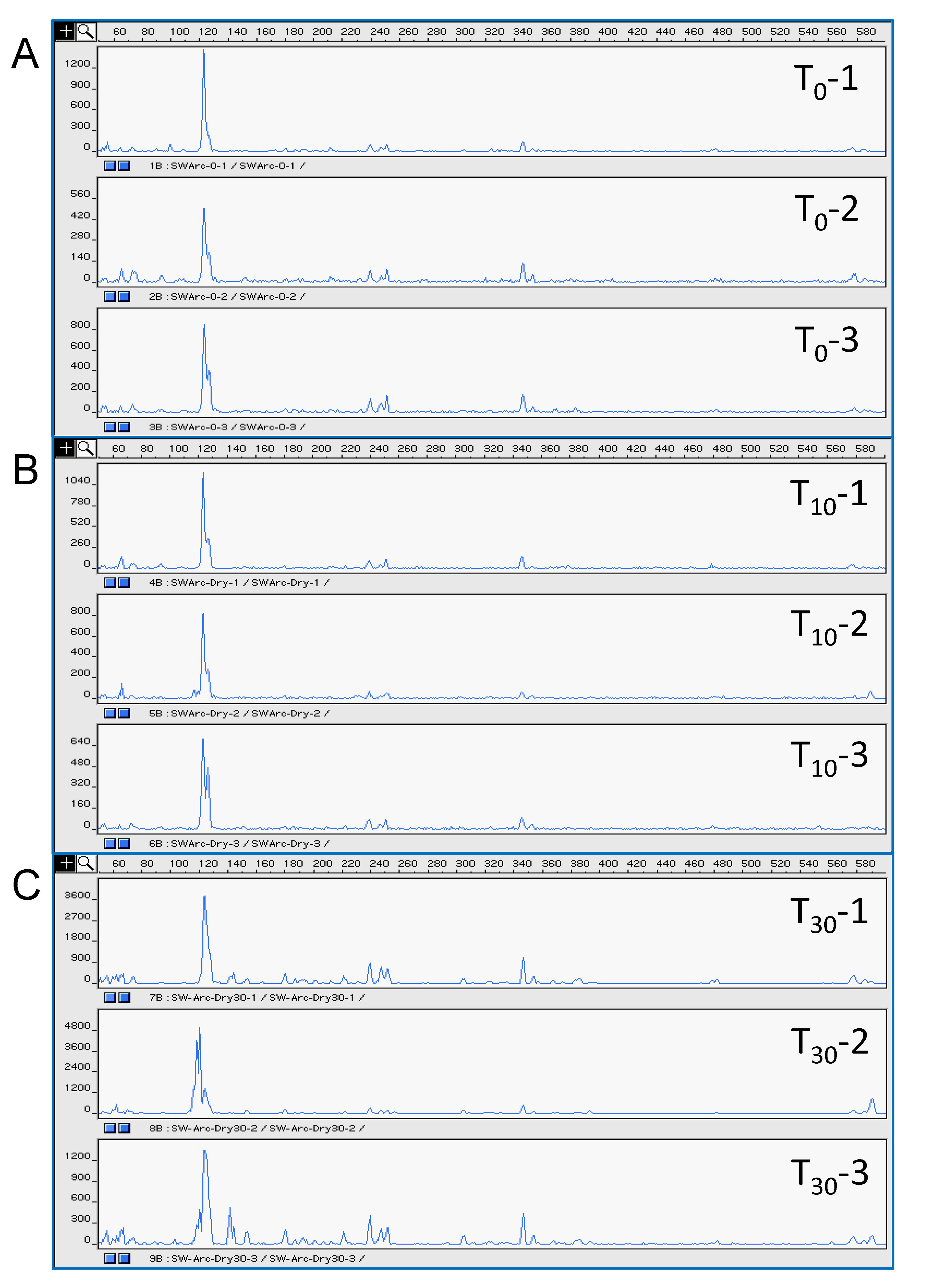

Supplement: S12 Fig — Panels display the T0 control samples (A) as well as those preserved for T10 and T30 days (B & C) respectively (n = 3 for each). (TIF) [file pone.0144686.s012.tif]

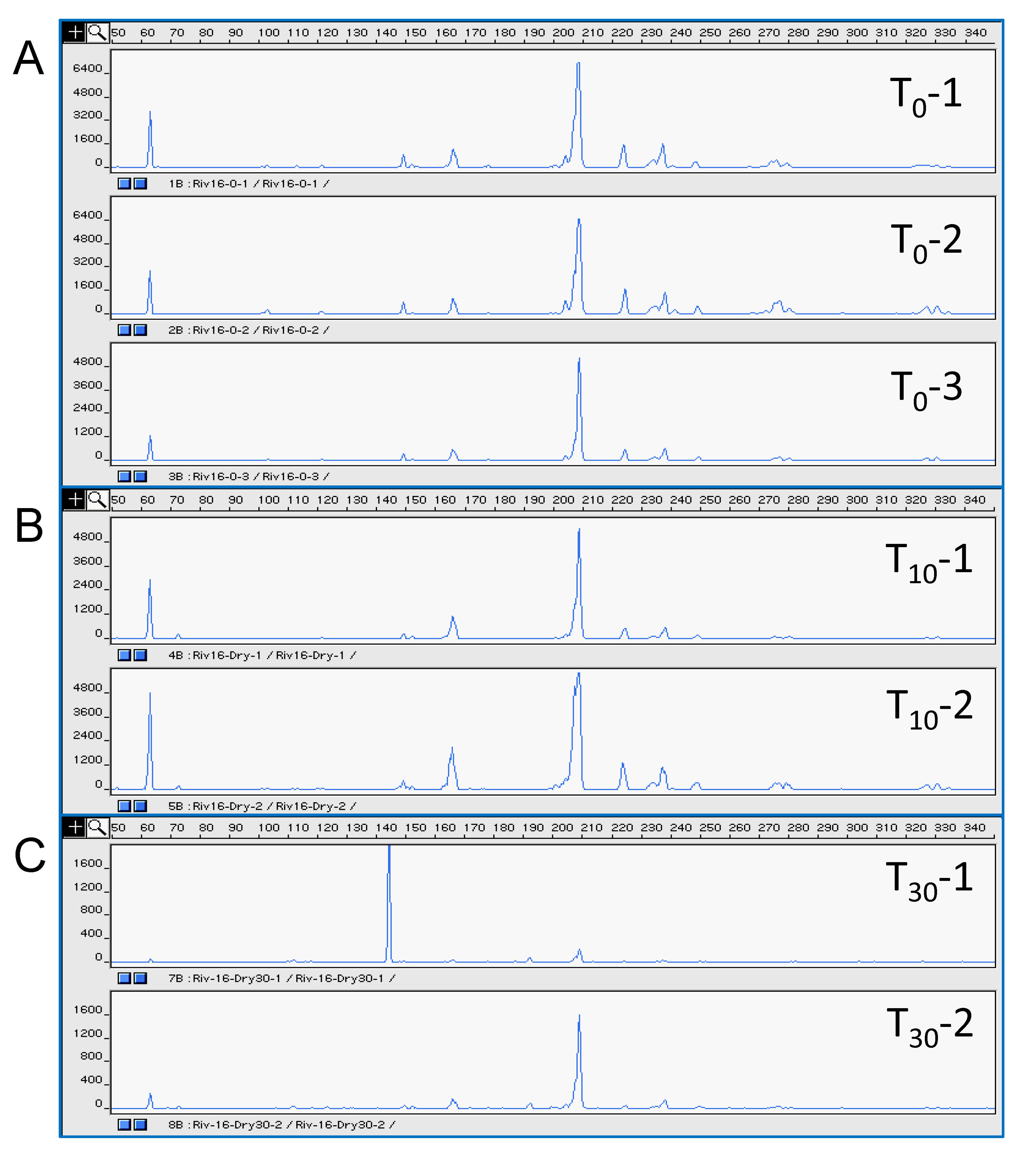

Supplement: S13 Fig — Panels display the T0 control samples (A) as well as those preserved for T10 and T30 days (B & C) respectively (n = 3, control; n = 2 for 10 & 30d). (TIF) [file pone.0144686.s013.tif]

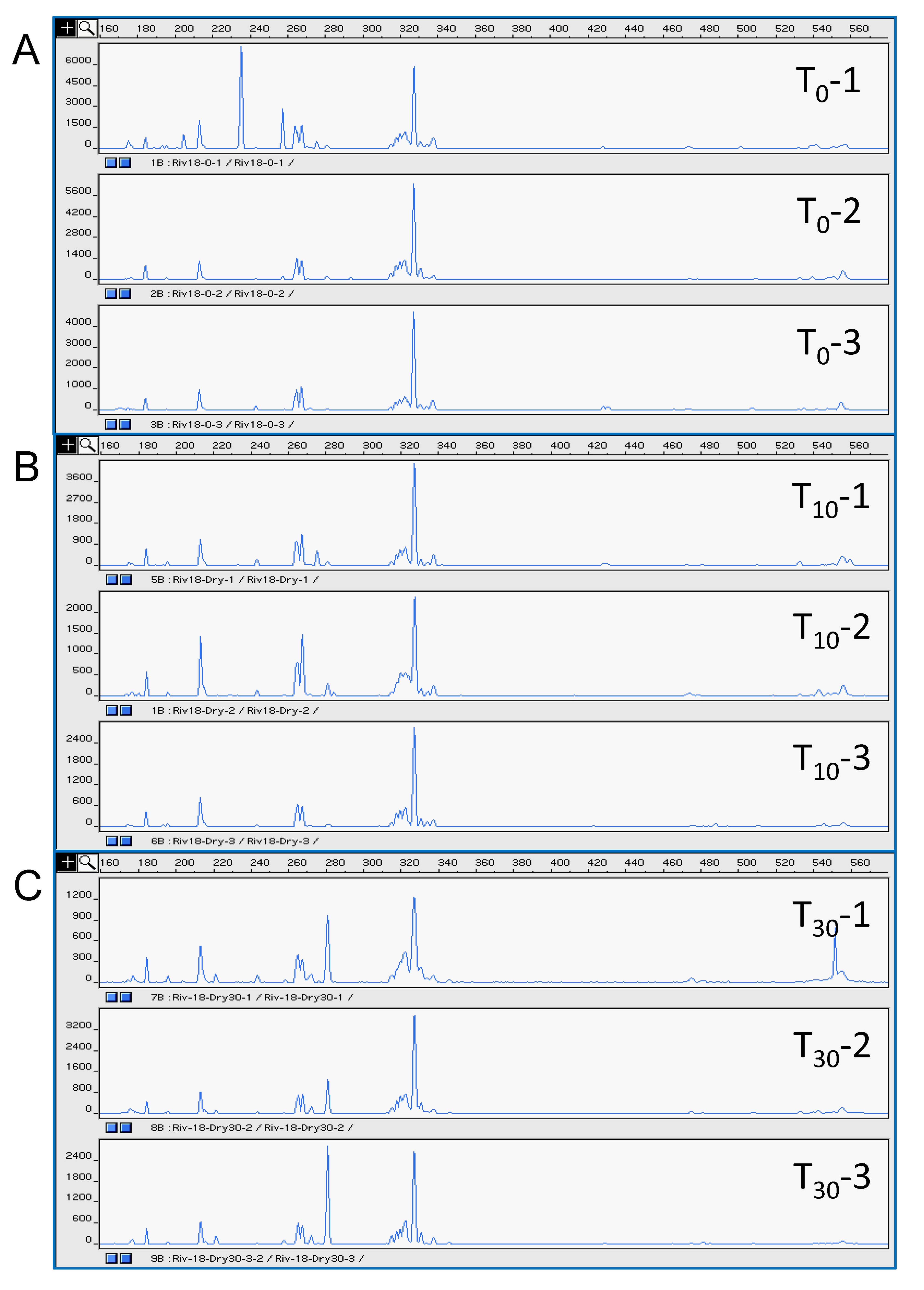

Supplement: S14 Fig — Panels display the T0 control samples (A) as well as those preserved for T10 and T30 days (B & C) respectively (n = 3 for each). (TIF) [file pone.0144686.s014.tif]
