# Supplementary material for: BioDry: An Inexpensive, Low-Power Method to Preserve Aquatic Microbial Biomass at Room Temperature
Source: PLoS One. 2015 Dec 28;10(12):e0144686. doi: 10.1371/journal.pone.0144686 (PMC4692454; doi:10.1371/journal.pone.0144686)
Supplement: S1 Table — (PDF) [file pone.0144686.s015.pdf]

S1 Table. Sequence Read Archive project, sample, experiment, and run accession numbers for 454 sequence libraries from the method verification samples – NCBI 454-pyrosequence accession numbers for the T_0 control and BioDried T_{15} and T_{30} samples.

	Accession Numbers				
**Treatment**	**Project**	**Study**	**Sample**	**Experiment**	**Run**
Time Zero	PRJNA278276	SRP058986	SRS951360	SRX1046596	SRR2048520
Time 15 days	PRJNA278276	SRP058986	SRS992110	SRX1096757	SRR2102054
Time 30 days	PRJNA278276	SRP058986	SRS992111	SRX1096756	SRR2102053
