# Supplementary material for: BioDry: An Inexpensive, Low-Power Method to Preserve Aquatic Microbial Biomass at Room Temperature
Source: PLoS One. 2015 Dec 28;10(12):e0144686. doi: 10.1371/journal.pone.0144686 (PMC4692454; doi:10.1371/journal.pone.0144686)
Supplement: S2 Table — (PDF) [file pone.0144686.s016.pdf]

S2 Table. Closest match species taxonomic ID for 454 sequences from the seawater samples – 454-pyrosequence species identification (closest match, percent similarity) and relative percent contribution of organisms detected in the T_0 control and BioDried T_{15} and T_{30} samples.

		% Contribution		
**SPECIES**	**Match (> %)**	**T_0**	**T_{15}**	**T_{30}**
Pseudoalteromonas spp.	98	37.3	40.6	37.0
Pseudoalteromonas ganghwensis	98	19.2	21.5	19.6
Phaeobacter gallaeciensis	99	4.3	2.9	4.4
Vibrio fortis	98	4.3	4.0	3.9
Pseudoalteromonas tetraodonis	97	4.0	3.9	4.4
Vibrio orientalis	98	3.5	3.5	3.3
Pseudoalteromonas denitrificans	97	3.4	2.8	3.6
GN02 (candidate division)	97	3.0	1.7	2.5
Marinobacterium spp.	97	2.1	2.1	1.8
Vibrio harveyi	99	2.0	1.8	1.5
Photobacterium spp.	96	1.2	1.0	1.1
Polaribacter spp.	96	1.1	1.0	1.1
Colwellia spp.	97	1.1	1.2	1.3
Vibrio proteolyticus	98	1.0	1.0	0.9
Pseudoalteromonas viridis	98	1.0	1.0	0.9
Phaeobacter spp.	97	0.9	0.8	1.1
Pseudoalteromonas porphyrae	94	0.8	1.2	0.7
Nautella italica	98	0.6	0.3	0.6
Oceanospirillum spp.	97	0.5	0.5	0.4
Roseovarius spp.	98	0.5	0.3	0.5
Roseobacter denitrificans	97	0.5	0.5	0.4

Vibrio shilonii	95	0.5	0.4	0.4
Thalassococcus spp.	98	0.4	0.4	0.4
Arcobacter spp.	98	0.4	0.2	0.4
Pseudoalteromonas elyakovii	97	0.3	0.3	0.2
Oleibacter spp.	95	0.3	0.2	0.2
Photobacterium damselae	99	0.3	0.2	0.2
Azospira spp.	91	0.2	0.1	0.4
Amphritea spp.	97	0.2	0.2	0.3
Psychroserpens spp.	97	0.2	0.2	0.2
Vibrio ichthyoenteri	98	0.2	0.2	0.2
Flavobacteriales bacterium	99	0.2	0.1	0.1
Shewanella spp.	95	0.2	0.2	0.1
Vibrio tapetis	97	0.2	0.1	0.1
Vibrio spp.	96	0.2	0.2	0.1
Loktanella spp.	97	0.1	0.1	0.2
Marinovum spp.	97	0.1	0.1	0.1
Marinobacterium jannaschii	99	0.1	0.1	0.2
Vibrio parahaemolyticus	98	0.1	0.1	0.1
Tenacibaculum spp.	92	0.1	0.1	0.0
Kordiimonas spp.	97	0.1	0.1	0.1
Pseudoalteromonas gracilis	97	0.1	0.2	0.1
Shewanella benthica	94	0.1	<0.05	<0.05
Pseudoalteromonas carrageenovora	97	0.1	0.2	0.1
Pseudoalteromonas rubra	96	0.1	0.1	0.1
Photobacterium rosenbergii	99	0.1	0.1	0.1
Pseudoalteromonas ruthenica	95	0.1	0.1	0.1
Neptunomonas spp.	96	0.1	0.1	0.1

Shewanella marinintestina	99	0.1	0.1	0.1
Owenweeksia spp.	98	0.1	0.1	0.2
Gilvibacter spp.	94	0.1	<0.05	0.1
Shewanella algae	99	0.1	0.1	<0.05
Tenacibaculum soleae	98	0.1	0.1	0.1
Yeosuana spp.	92	0.1	0.1	0.1
Pseudoalteromonas luteoviolacea	96	0.1	0.1	0.1
Ulvibacter spp.	94	0.1	0.1	0.1
Colwellia maris	93	0.1	<0.05	0.1
Marinomonas arenicola	94	0.1	0.1	0.0
Thalassomonas spp.	92	0.1	0.1	0.1
Teredinibacter spp.	98	0.1	<0.05	<0.05
Vibrio vulnificus	94	0.1	0.1	<0.05
Winogradskyella spp.	97	0.1	<0.05	<0.05
Olleya spp.	92	<0.05	<0.05	<0.05
Thalassomonas viridans	95	<0.05	<0.05	<0.05
Tropicibacter spp.	99	<0.05	<0.05	0.1
Alteromonas spp.	95	<0.05	<0.05	0.1
Pseudoalteromonas byunsanensis	94	<0.05	<0.05	<0.05
Crocinitomix spp.	99	<0.05	<0.05	<0.05
Photobacterium phosphoreum	97	<0.05	<0.05	<0.05
Pseudospirillum spp.	97	<0.05	<0.05	<0.05
Tenacibaculum aestuarii	93	<0.05	<0.05	<0.05
Vibrio agarivorans	94	<0.05	<0.01	<0.01
Vibrio rumoiensis	93	<0.05	<0.05	<0.05
Aeromonas spp.	93	<0.05	<0.05	<0.05
Colwellia rossensis	94	<0.05	<0.05	<0.05
Lutibacter spp.	92	<0.05	<0.05	<0.05

Moritella viscosa	97	<0.05	<0.05	0.1
Sufflavibacter spp.	98	<0.05	<0.01	<0.05
Marinomonas spp.	97	<0.05	<0.05	0.1
Ruegeria spp.	99	<0.05	<0.01	0.1
Shewanella livingstonensis	95	<0.05	<0.05	<0.01
Sulfitobacter spp.	98	<0.05	<0.05	<0.05
Teredinibacter proteobacteria	98	<0.05	0.1	<0.05
Vibrio metschnikovii	97	<0.05	<0.05	<0.05
Aliivibrio vibrio fischeri	94	<0.05	<0.01	<0.05
Alteromonas marina	99	<0.05	<0.05	0.1
Colwellia psychrerythraea	94	<0.05	<0.05	<0.05
Maritimimonas spp.	92	<0.05	<0.01	<0.01
Nisaea spp.	88	<0.05	<0.05	<0.05
Pseudoalteromonas haloplanktis	96	<0.05	0.1	<0.05
Rhodovulum spp.	98	<0.05	<0.05	0.0
Salinihabitans spp.	97	<0.05	<0.01	<0.05
Shewanella aquimarina	97	<0.05	<0.05	<0.05
Thalassomonas agarivorans	95	<0.05	<0.05	0.0
Aliivibrio logei	94	<0.05	<0.05	<0.05
Marinosulfonomonas spp.	97	<0.05	<0.05	<0.05
Nautella spp.	96	<0.05	<0.01	<0.05
Photobacterium lipolyticum	92	<0.05	<0.01	<0.01
Dasania spp.	97	<0.05	<0.01	0.0
Formosa spp.	93	<0.05	<0.01	<0.01
Oceanicola spp.	96	<0.05	<0.05	0.0
Oceaniserpentilla spp.	99	<0.05	<0.01	<0.05
Rheinheimera spp.	92	<0.05	<0.01	<0.05
Vibrio gallicus	95	<0.05	<0.05	<0.05

Candidatus pelagibacter ubique	99	<0.05	0.0	0.1
Kangiella koreensis	97	<0.05	<0.01	0.0
Marixanthomonas spp.	92	<0.05	0.0	<0.05
Thalassospira spp.	97	<0.05	<0.01	<0.05
Cohaesibacter spp.	96	<0.01	0.0	<0.01
Litoricola spp.	85	<0.01	<0.01	<0.05
Nitrosococcus spp.	93	<0.01	<0.01	0.0
Oleiphilus spp.	92	<0.01	<0.05	0.0
Pseudoalteromonas citrea	95	<0.01	<0.01	<0.05
Roseobacter spp.	96	<0.01	<0.05	<0.05
Shewanella woodyi	88	<0.01	<0.01	<0.05
Flavobacterium spp.	96	<0.01	<0.05	<0.05
Fluviicola spp.	92	<0.01	<0.01	0.0
Jannaschia donghaensis	87	<0.01	<0.01	0.0
Labrenzia aggregata	99	<0.01	0.0	<0.01
Melitea spp.	93	<0.01	<0.01	0.0
Oleispira spp.	97	<0.01	<0.05	<0.05
Pelagibius spp.	91	<0.01	<0.01	0.0
Rhodobium spp.	90	<0.01	<0.01	<0.01
Thalassospira xiamenensis	99	<0.01	<0.01	<0.05
Acidimicrobiales spp.	88	0.0	<0.01	<0.01
Balneatrix spp.	94	0.0	<0.01	<0.01
Psychroserpens burtonensis	95	0.0	<0.01	<0.05
Salinisphaera spp.	90	0.0	<0.01	<0.01
Thalassolituus spp.	92	0.0	<0.01	<0.05
Thiothrix spp.	99	0.0	<0.01	<0.01
