# Supplementary material for: BioDry: An Inexpensive, Low-Power Method to Preserve Aquatic Microbial Biomass at Room Temperature
Source: PLoS One. 2015 Dec 28;10(12):e0144686. doi: 10.1371/journal.pone.0144686 (PMC4692454; doi:10.1371/journal.pone.0144686)
Supplement: S3 Table — (PDF) [file pone.0144686.s017.pdf]

S3 Table. Bray-Curtis similarity index of the DNA 454-pyrosequence analysis comparing the bacterial community structures of the T_0, T_{15}, T_{30}.

	T-0	T-15	T-30
**T-0**	100.0	93.2	96.3
**T-15**	93.2	100.0	92.2
**T-30**	96.3	92.2	100.0
