# Supplementary material for: BioDry: An Inexpensive, Low-Power Method to Preserve Aquatic Microbial Biomass at Room Temperature
Source: PLoS One. 2015 Dec 28;10(12):e0144686. doi: 10.1371/journal.pone.0144686 (PMC4692454; doi:10.1371/journal.pone.0144686)
Supplement: S4 Table — (PDF) [file pone.0144686.s018.pdf]

S4 Table. Bray-Curtis similarity index of the DNA-TRFLP analysis comparing the bacterial community structures of all T_0, T_{15}, and T_{30} replicates from the method verification tests.

	**0**	**15**	**30**
**0**	100.0	85.2	86.4
**15**	85.2	100.0	85.2
**30**	86.4	85.2	100.0
