# Supplementary material for: BioDry: An Inexpensive, Low-Power Method to Preserve Aquatic Microbial Biomass at Room Temperature
Source: PLoS One. 2015 Dec 28;10(12):e0144686. doi: 10.1371/journal.pone.0144686 (PMC4692454; doi:10.1371/journal.pone.0144686)
Supplement: S5 Table — (PDF) [file pone.0144686.s019.pdf]

S5 Table. Bray-Curtis similarity index of the RNA-TRFLP analysis comparing the bacterial community structures of all T_0, T_{15}, and T_{30} replicates from the method verification tests.

	T0-1	T0-2	T15-1	T15-2	T15-3	T15-4	T15-5	T15-6	T15-7	T15-8	T30-1	T30-2	T30-3	T30-4	T30-5	T30-6	T30-7
**T0-1**	100.0	86.8	83.5	80.7	89.3	90.2	89.6	90.2	89.3	87.3	80.4	86.4	83.6	84.7	84.6	84.0	86.8
**T0-2**	86.8	100.0	93.2	90.9	88.6	88.4	90.9	90.4	93.0	88.9	90.5	91.1	91.4	88.8	91.0	87.2	90.0
**T15-1**	83.5	93.2	100.0	94.4	87.4	87.2	89.2	89.8	90.1	89.6	95.6	90.2	91.2	87.3	92.0	89.9	89.2
**T15-2**	80.7	90.9	94.4	100.0	85.1	84.2	86.8	87.2	88.3	86.3	95.1	87.9	88.9	84.4	89.4	87.1	86.5
**T15-3**	89.3	88.6	87.4	85.1	100.0	96.3	95.9	96.2	94.8	94.3	85.8	93.5	92.0	91.7	92.0	93.0	94.2
**T15-4**	90.2	88.4	87.2	84.2	96.3	100.0	96.3	95.2	94.1	94.0	85.1	91.3	89.8	90.7	90.0	91.4	93.0
**T15-5**	89.6	90.9	89.2	86.8	95.9	96.3	100.0	97.0	97.0	95.5	87.7	94.0	92.0	91.5	93.2	93.6	95.5
**T15-6**	90.2	90.4	89.8	87.2	96.2	95.2	97.0	100.0	96.7	95.5	88.1	93.4	91.5	90.8	92.9	92.7	94.0
**T15-7**	89.3	93.0	90.1	88.3	94.8	94.1	97.0	96.7	100.0	94.4	88.6	95.3	92.7	92.8	94.4	92.0	94.6
**T15-8**	87.3	88.9	89.6	86.3	94.3	94.0	95.5	95.5	94.4	100.0	88.9	93.1	91.4	91.2	92.8	95.1	94.7
**T30-1**	80.4	90.5	95.6	95.1	85.8	85.1	87.7	88.1	88.6	88.9	100.0	89.4	90.7	86.6	91.2	89.6	88.6
**T30-2**	86.4	91.1	90.2	87.9	93.5	91.3	94.0	93.4	95.3	93.1	89.4	100.0	95.5	95.0	96.2	94.8	96.5
**T30-3**	83.6	91.4	91.2	88.9	92.0	89.8	92.0	91.5	92.7	91.4	90.7	95.5	100.0	93.5	95.1	93.4	93.4
**T30-4**	84.7	88.8	87.3	84.4	91.7	90.7	91.5	90.8	92.8	91.2	86.6	95.0	93.5	100.0	92.4	92.0	94.4
**T30-5**	84.6	91.0	92.0	89.4	92.0	90.0	93.2	92.9	94.4	92.8	91.2	96.2	95.1	92.4	100.0	94.7	94.2
**T30-6**	84.0	87.2	89.9	87.1	93.0	91.4	93.6	92.7	92.0	95.1	89.6	94.8	93.4	92.0	94.7	100.0	95.7
**T30-7**	86.8	90.0	89.2	86.5	94.2	93.0	95.5	94.0	94.6	94.7	88.6	96.5	93.4	94.4	94.2	95.7	100.0
