# Supplementary material for: BioDry: An Inexpensive, Low-Power Method to Preserve Aquatic Microbial Biomass at Room Temperature
Source: PLoS One. 2015 Dec 28;10(12):e0144686. doi: 10.1371/journal.pone.0144686 (PMC4692454; doi:10.1371/journal.pone.0144686)
Supplement: S6 Table — (PDF) [file pone.0144686.s020.pdf]

S6 Table. Bray-Curtis similarity index of the RNA-TRFLP analysis comparing the seawater bacterial community structures of all T_0, T_{10}, and T_{30} replicates from the field tests.

	T0-1	T0-2	T0-3	T10-1	T10-2	T10-3	T30-1	T30-2	T30-3
**T0-1**	100.0	92.8	83.1	91.7	89.1	89.0	65.6	66.1	70.4
**T0-2**	92.8	100.0	83.1	93.5	91.9	89.6	66.8	64.1	70.8
**T0-3**	83.1	83.1	100.0	84.1	84.3	81.3	58.6	61.4	62.0
**T10-1**	91.7	93.5	84.1	100.0	91.0	89.8	66.7	64.9	69.9
**T10-2**	89.1	91.9	84.3	91.0	100.0	86.4	62.7	60.9	64.7
**T10-3**	89.0	89.6	81.3	89.8	86.4	100.0	71.3	67.8	74.0
**T30-1**	65.6	66.8	58.6	66.7	62.7	71.3	100.0	80.4	89.8
**T30-2**	66.1	64.1	61.4	64.9	60.9	67.8	80.4	100.0	82.1
**T30-3**	70.4	70.8	62.0	69.9	64.7	74.0	89.8	82.1	100.0
