# Supplementary material for: BioDry: An Inexpensive, Low-Power Method to Preserve Aquatic Microbial Biomass at Room Temperature
Source: PLoS One. 2015 Dec 28;10(12):e0144686. doi: 10.1371/journal.pone.0144686 (PMC4692454; doi:10.1371/journal.pone.0144686)
Supplement: S7 Table — (PDF) [file pone.0144686.s021.pdf]

S7 Table. Bray-Curtis similarity index of the RNA-TRFLP analysis comparing the seawater eukaryotic community structures of all T_0, T_{10}, and T_{30} replicates from the field tests.

	T0-1	T0-2	T0-3	T10-1	T10-2	T10-3	T30-1	T30-2	T30-3
**T0-1**	100.0	88.6	88.5	87.1	88.6	92.1	58.6	56.6	56.3
**T0-2**	88.6	100.0	93.3	90.6	82.1	86.3	63.8	62.1	61.5
**T0-3**	88.5	93.3	100.0	89.1	80.7	85.5	63.7	62.0	62.5
**T10-1**	87.1	90.6	89.1	100.0	85.8	89.5	59.9	58.3	58.0
**T10-2**	88.6	82.1	80.7	85.8	100.0	91.2	51.2	49.7	49.4
**T10-3**	92.1	86.3	85.5	89.5	91.2	100.0	59.5	56.9	56.7
**T30-1**	58.6	63.8	63.7	59.9	51.2	59.5	100.0	90.6	91.2
**T30-2**	56.6	62.1	62.0	58.3	49.7	56.9	90.6	100.0	95.1
**T30-3**	56.3	61.5	62.5	58.0	49.4	56.7	91.2	95.1	100.0
