# Supplementary material for: BioDry: An Inexpensive, Low-Power Method to Preserve Aquatic Microbial Biomass at Room Temperature
Source: PLoS One. 2015 Dec 28;10(12):e0144686. doi: 10.1371/journal.pone.0144686 (PMC4692454; doi:10.1371/journal.pone.0144686)
Supplement: S8 Table — (PDF) [file pone.0144686.s022.pdf]

**S8 Table. Bray-Curtis similarity index of the RNA-TRFLP analysis comparing the river bacterial community structures of all $T_0$, $T_{10}$, and $T_{30}$ replicates from the field tests.**

| | T0-2 | T0-3 | T10-1 | T10-2 | T30-1 | T30-2 | T30-3 |
|---|---|---|---|---|---|---|---|
| **T0-2** | 100.0 | 79.2 | 71.8 | 73.1 | 68.3 | 36.8 | 70.3 |
| **T0-3** | 79.2 | 100.0 | 77.7 | 71.1 | 83.3 | 37.2 | 77.3 |
| **T10-1** | 71.8 | 77.7 | 100.0 | 78.3 | 88.0 | 36.9 | 77.3 |
| **T10-2** | 73.1 | 71.1 | 78.3 | 100.0 | 74.6 | 37.2 | 66.8 |
| **T30-1** | 68.3 | 83.3 | 88.0 | 74.6 | 100.0 | 37.2 | 83.8 |
| **T30-2** | 36.8 | 37.2 | 36.9 | 37.2 | 37.2 | 100.0 | 39.3 |
| **T30-3** | 70.3 | 77.3 | 77.3 | 66.8 | 83.8 | 39.3 | 100.0 |
