# Supplementary material for: BioDry: An Inexpensive, Low-Power Method to Preserve Aquatic Microbial Biomass at Room Temperature
Source: PLoS One. 2015 Dec 28;10(12):e0144686. doi: 10.1371/journal.pone.0144686 (PMC4692454; doi:10.1371/journal.pone.0144686)
Supplement: S9 Table — (PDF) [file pone.0144686.s023.pdf]

S9 Table. Bray-Curtis similarity index of the RNA-TRFLP analysis comparing the river eukaryotic community structures of all T_0, T_{10}, and T_{30} replicates from the field tests.

	18-0-1	18-0-2	18-0-3	18-10-1	18-10-2	18-10-3	18-30-1	18-30-2	18-30-3
**18-0-1**	100.0	67.5	74.6	72.4	70.2	69.2	64.2	68.8	66.2
**18-0-2**	67.5	100.0	82.1	84.2	73.0	86.7	76.7	83.3	70.5
**18-0-3**	74.6	82.1	100.0	86.5	77.8	82.8	71.8	75.9	67.1
**18-10-1**	72.4	84.2	86.5	100.0	80.0	87.6	76.3	80.0	69.4
**18-10-2**	70.2	73.0	77.8	80.0	100.0	78.4	72.3	72.1	64.6
**18-10-3**	69.2	86.7	82.8	87.6	78.4	100.0	74.9	81.7	69.9
**18-30-1**	64.2	76.7	71.8	76.3	72.3	74.9	100.0	83.0	81.8
**18-30-2**	68.8	83.3	75.9	80.0	72.1	81.7	83.0	100.0	80.4
**18-30-3**	66.2	70.5	67.1	69.4	64.6	69.9	81.8	80.4	100.0
