# Supplementary material for: BioDry: An Inexpensive, Low-Power Method to Preserve Aquatic Microbial Biomass at Room Temperature
Source: PLoS One. 2015 Dec 28;10(12):e0144686. doi: 10.1371/journal.pone.0144686 (PMC4692454; doi:10.1371/journal.pone.0144686)
Supplement: S10 Table — (PDF) [file pone.0144686.s024.pdf]

S10 Table. Bray-Curtis similarity index of the RNA-TRFLP analysis comparing the seawater archaeal community structures of all T_0, T_{10}, and T_{30} replicates from the field tests.

	T0-1	T0-2	T0-3	T10-1	T10-2	T10-3	T30-1	T30-2	T30-3
**T0-1**	100.0	53.4	53.0	46.3	36.3	29.9	46.8	28.9	49.0
**T0-2**	53.4	100.0	85.9	79.9	68.2	62.1	68.9	36.8	55.8
**T0-3**	53.0	85.9	100.0	82.0	74.2	67.5	69.8	35.8	56.6
**T10-1**	46.3	79.9	82.0	100.0	79.8	69.4	66.9	33.4	52.4
**T10-2**	36.3	68.2	74.2	79.8	100.0	73.1	57.3	40.0	49.4
**T10-3**	29.9	62.1	67.5	69.4	73.1	100.0	53.3	31.1	42.4
**T30-1**	46.8	68.9	69.8	66.9	57.3	53.3	100.0	46.1	73.4
**T30-2**	28.9	36.8	35.8	33.4	40.0	31.1	46.1	100.0	61.8
**T30-3**	49.0	55.8	56.6	52.4	49.4	42.4	73.4	61.8	100.0
